# Microorganisms in Confined Habitats: Microbial Monitoring and Control of Intensive Care Units, Operating Rooms, Cleanrooms and the International Space Station

**DOI:** 10.3389/fmicb.2016.01573

**Published:** 2016-10-13

**Authors:** Maximilian Mora, Alexander Mahnert, Kaisa Koskinen, Manuela R. Pausan, Lisa Oberauner-Wappis, Robert Krause, Alexandra K. Perras, Gregor Gorkiewicz, Gabriele Berg, Christine Moissl-Eichinger

**Affiliations:** ^1^Department for Internal Medicine, Medical University of Graz, GrazAustria; ^2^Institute of Environmental Biotechnology, Graz University of Technology, GrazAustria; ^3^BioTechMed-Graz, GrazAustria; ^4^Department of Pathology, Medical University of Graz, GrazAustria; ^5^Department for Microbiology, University of Regensburg, RegensburgGermany

**Keywords:** microbiome, built environment, indoor, confined habitat, microorganisms

## Abstract

Indoor environments, where people spend most of their time, are characterized by a specific microbial community, the indoor microbiome. Most indoor environments are connected to the natural environment by high ventilation, but some habitats are more confined: intensive care units, operating rooms, cleanrooms and the international space station (ISS) are extraordinary living and working areas for humans, with a limited exchange with the environment. The purposes for confinement are different: a patient has to be protected from infections (intensive care unit, operating room), product quality has to be assured (cleanrooms), or confinement is necessary due to extreme, health-threatening outer conditions, as on the ISS. The ISS represents the most secluded man-made habitat, constantly inhabited by humans since November 2000 – and, inevitably, also by microorganisms. All of these man-made confined habitats need to be microbiologically monitored and controlled, by e.g., microbial cleaning and disinfection. However, these measures apply constant selective pressures, which support microbes with resistance capacities against antibiotics or chemical and physical stresses and thus facilitate the rise of survival specialists and multi-resistant strains. In this article, we summarize the available data on the microbiome of aforementioned confined habitats. By comparing the different operating, maintenance and monitoring procedures as well as microbial communities therein, we emphasize the importance to properly understand the effects of confinement on the microbial diversity, the possible risks represented by some of these microorganisms and by the evolution of (antibiotic) resistances in such environments – and the need to reassess the current hygiene standards.

## Introduction

Nowadays, people spend most of their time indoors (up to 90% in industrialized countries; Hppe and Martinac, 1998). In particular, the process of increasing urbanization has created new types of microbiome settings that surround us in our living and work space, such as air conditioned residences and highly populated offices. The microbiome of a built environment is determined by numerous parameters, such as geographic location, type of usage, architectural design, ventilation and occupancy, but mainly by the living inhabitants (humans, animals, and plants), as the major source of microorganisms (Califf et al., 2014; Mahnert et al., 2015a; Meadow et al., 2015). For example the human body is a holobiont and thus the home of billions of microbes. Every second of our lives, we interact with microorganisms that support our life and health. This cohabitation has evolved over 1000s of years, and is characterized by a balanced interaction of three domains of life, namely the Archaea, Bacteria, and Eukaryota (Parfrey et al., 2011; Human Microbiome Project Consortium, 2012; Probst et al., 2013; Gaci et al., 2014). It was calculated that a human body can emit up to 3.7 × 10^7^ bacterial and 7.3 × 10^6^ fungal genome copies per hour (Qian et al., 2012).

**FIGURE 1:**
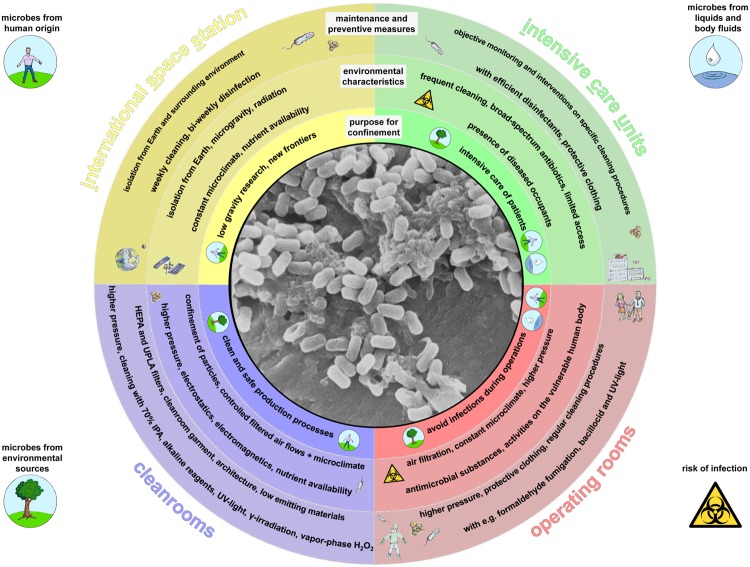
**Graphical display of the confined habitats addressed in this review.** Outer rings summarize environmental conditions of the purpose for confinement, some characteristics of each confined environment and overall maintenance and preventive measures in respective built environments. Potential contamination and infection sources are highlighted by small graphics. Inner circle: Bacillus spores, scanning electron micrograph.

In the study by Ruiz-Calderon et al. (2016) different housing types were analyzed with respect to the indoor microbial community, starting with jungle villages to highly urbanized living areas in Manaus. Although all of the analyzed living areas were well ventilated, the housings of higher urbanization level were characterized by a reduced influence of the outer, natural environmental microbiome whereas the portion of human-associated microorganisms was substantially increased. As a logical conclusion, more confined environments, with less or no contact to the outdoor environment, should be totally dominated by human associated microorganisms. There are many reasons that necessitate stricter confinement for living and work environments than is typical for most people. For the purposes of this review, we are interested in confined habitats as defined by human-populated environments restricted by a number of parameters. The parameters are a restriction of area and space, and restrictions of physical, chemical and biological exchange with the surrounding, natural environment. Such confined habitats include areas such as intensive care units (ICUs) and operating rooms, where patients need to be protected from infection; cleanrooms, where the quality of products needs to be assured; and the ISS, which is encapsulated due to life-threatening environmental conditions. A summary of the characteristics of the confined habitats addressed in this review is given in **Figure 1**.

All these environments require microbiological monitoring, and control, since they harbor their own, possibly adapted, microbial community, which is greatly influenced by the maintenance regime.

In this review, we detail the setting, architecture, and control measures of such environments, which influence the internal microbiome tremendously. We hypothesize that all these environments have parameters in common, which shape, in a similar way, the inhabiting microbial community – with a potential effect on humans living and/or working in these areas.

## The Microbiology of Intensive Care Units

### Intensive Care Units and Hospital Acquired Infections

Intensive care units are special departments in hospitals that provide intensive medical care for patients suffering from severe and life-threatening diseases or injuries. These units can be divided into several categories, including neonatal ICUs, pediatric ICUs, psychiatric ICUs, cardiac ICUs, medical ICUs, neurological ICUs, trauma ICUs, and surgical ICUs. Depending on the underlying disease, duration of stay and treatment in ICUs, patients may show higher susceptibility for hospital-acquired infections (HAIs) than healthy individuals due to an overall weak condition, immunosuppression, or disrupted physiological barriers. ICUs are considered potential reservoirs for (opportunistic) pathogenic microbial strains (Russotto et al., 2015). These microorganisms may thrive on the medical equipment, in other patients, personnel, and the surrounding environment of the hospital (Gastmeier et al., 2007). HAIs are a serious problem worldwide: in the United States, HAIs are the sixth leading cause of death, killing more people than diabetes or influenza combined (Anderson and Smith, 2005; Klevens et al., 2007), and similar results have been reported from Europe as well (Peleg and Hooper, 2010). For instance, Vincent et al. (1995) have estimated the risk for gaining a nosocomial infection in a European ICU to be 45%. In general, the risk of acquiring pathogenic infection, in hospital environments is higher than in other environments, and the course of an infection is more often fatal (Centers for Disease and Prevention, 2002, 2010; Klevens et al., 2007).

Already in the 1980’s, specialists in infectious diseases detected that patients in ICUs are infected by nosocomial bacteria, as e.g., *Pseudomonas aeruginosa* and *Acinetobacter baumannii*, considerably more often than patients in other wards in the hospital (Donowitz et al., 1982). Many factors contribute to the increased infection rate in ICUs, including the underlying disease of the patient, the length of the hospitalization, frequency of contact with medical personnel, the number of colonized or infected patients in the same ward, ICU structure (single bed vs. double bed rooms), and the lack of compliance with existing infection prevention guidelines (Siegel et al., 2007). Even the season affects the incidence: in wintertime the risk of acquiring a HAI is smaller compared to other seasons (Schröder et al., 2015). Patient groups that are most often affected are the elderly, premature infants and patients suffering from immunodeficiency (Unahalekhaka, 2011); in the latter, even non-virulent bacteria may cause serious infection and death (Poza et al., 2012).

The risk of infection is increased by invasive, clinically necessary procedures (like insertion of catheters), but also from architectural properties of the hospital environments (such as ventilation systems; Unahalekhaka, 2011) or deficient hygiene procedures. For instance, significantly higher risk for the acquisition of antibiotic resistant microorganisms was observed when newly arrived patients were placed in rooms that were previously occupied by carriers, despite terminal cleaning of the ICU bed space (Huang et al., 2006; Russotto et al., 2015). This transfer was confirmed by another study, reporting that the infection of the previous room occupant was the most important independent risk factor for infection with *Pseudomonas aeruginosa* and *Acinetobacter baumannii*, two bacteria causing nosocomial infections (Nseir et al., 2011). The majority of the HAIs is believed to be transmitted directly from patient to patient, but increasing evidence demonstrates that also the medical personnel as well as the clinical environment (i.e., surfaces and equipment) often are a source of infection (Tringe and Hugenholtz, 2008; Caporaso et al., 2012; Passaretti et al., 2013; Salgado et al., 2013). One major vector for cross-contamination are hands of medical personnel, contributing to approximately 20–40% of nosocomial infections (Agodi et al., 2007; Weber et al., 2010). Since infected patients themselves act as a source of microorganisms, frequently touched surfaces close to the patient were heavily contaminated (Wertheim et al., 2005; Pittet et al., 2006). Specifically, Salgado et al. (2013) observed that the risk of acquiring a nosocomial infection increased significantly when the total microbial burden exceeded 500 CFU/100 cm^2^.

The link of invasive equipment and the emergence of nosocomial infections has clearly been shown. However, there is also evidence of non-invasive devices to cause ICU outbreaks. Especially, electrical equipment and devices that are difficult to clean (irregular shape, no cleaning regime) have been reported as a source for infection (Russotto et al., 2015).

Hospital textiles are another potential source of HAIs. These textiles are usually reusable and include uniforms, bed linen and pajamas, as well as privacy curtains and protective clothing of health care personnel. The liberation and dispersal of bioaerosols and fomites from textiles takes place during handling of soiled textiles that have been used by or have been in close contact with an infected patient. It has been shown that antibiotic resistant *Staphylococcus* strains can aerosolize from bed linen during routine handling of bedding and be transmitted via air (Handorean et al., 2015). However, microbial transfer from textiles can be easily prevented by proper laundry procedures (Fijan and Turk, 2012).

### The ICU Microbiome

Previous studies have shown that pathogenic bacteria, such as *Staphylococcus aureus*, various *Enterococcus* species, *Escherichia coli, Pseudomonas aeruginosa, Klebsiella pneumonia*, different *Enterobacter* species, *Acinetobacter baumannii* and *Klebsiella oxytoca* are, despite efficient cleaning procedures and disinfectants, commonly found on surfaces such as stethoscopes (Marinella et al., 1997), electronic thermometers (Livornese et al., 1992), and other equipment routinely used in hospitals (Myers, 1978; Schabrun et al., 2006; Safdar et al., 2012).

Bacteria living in diverse communities at ICUs include pathogenic strains, opportunistic pathogens, as well as harmless and beneficial bacteria. Bacteria found in ICU environments are typically human associated and, due to confinement and strict cleaning procedures, less diverse than indoor environments with unlimited and uncontrolled access. In addition to the above mentioned common hospital pathogens, several genera of opportunistic pathogens have been detected in hospital environments by cultivation and using next generation sequencing methods, including *Actinomyces, Burkholderia, Clostridium, Flavobacterium, Neisseria, Propionibacterium, Roseomonas, Streptococcus*, and *Vibrio* (e.g., Kim et al., 1981; Heeg et al., 1994; Triassi et al., 2006; Hewitt et al., 2013; Oberauner et al., 2013). Bacterial communities in different locations at an ICU vary in species composition and diversity. In general, objects and surfaces near patients, including textiles such as pajamas, bedlinen, pillows and mattresses, carry more human gut-, hair- and skin-associated bacteria like *Staphylococcus, Propionibacteria, Corynebacteria, Lactobacillus, Micrococcus and Streptococcus*, whereas floor and other sites with greater distance to the patient carry more environmental strains. In addition, the abundance of bacteria was higher if samples were taken close to the patient (Handorean et al., 2015; Hu et al., 2015). However, according to current knowledge, most of the detected bacteria are harmless or beneficial and include, for example, *Bradyrhizobium, Corynebacterium, Delftia, Lactobacillus, Melissococcus, Prevotella, Paracoccus, Sandaracinobacter*, and *Sphingobium* (Hewitt et al., 2013).

(Opportunistic) pathogenic bacteria are typically resistant to various stresses. Due to the extreme selective pressure that confinement and cleaning practices induce, microorganisms living in ICUs develop or acquire resistance mechanisms that allow them to survive in the presence of a vast range of antimicrobial agents used in cleaning and antibiotic treatment, to adapt to extremely low nutrient content, and to persist on dry surfaces for a long time (Poza et al., 2012). In particular biofilms (including multispecies biofilms (Fux et al., 2005)) can resist common cleaning protocols. Their cells, embedded in the matrix of a biofilm, are considerably more tolerant to desiccation, detergents and disinfectants than planktonic bacteria (Burmølle et al., 2006), making them a highly dangerous infection source for susceptible patients and a critical target for bacterial burden control (Kramer et al., 2006; Hu et al., 2015). The presence of these multispecies biofilms on various surfaces may contribute to the stability of harmful bacteria in ICUs. In a recent study, Hu et al. (2015) showed that these diverse biofilms can even tolerate terminal cleaning procedures of ICU facilities and harbor viable bacteria even after 1 year (Vickery et al., 2012). Biofilms have been detected in various locations in ICUs, including a box for sterile supplies, a privacy curtain, a glove box, a noticeboard, and catheters (Perez et al., 2014; Hu et al., 2015). According to Hu et al. (2015) up to 93% of studied surfaces carried bacterial biofilms. In addition, the biofilm lifestyle of microorganisms bears a high risk for horizontal gene transfer, consequent spreading of antibiotic resistance and high possibility for recurrence (Fux et al., 2005). Common examples of multidrug resistance (MDR) are methicillin-resistant *Staphylococcus aureus* (MRSA) and vancomycin-resistant enterococci (VRE) that are also typical components of the ICU microbiome. Often similar cellular mechanisms are used in virulence, antibiotic resistance and resistance to toxic compounds, such as cleaning agents (Daniels and Ramos, 2009; Beceiro et al., 2013).

### The Microbiome Of Neonatal ICUs

Neonatal intensive-care units (NICUs) are specialized in the treatment of seriously health-threatened or prematurely born infants. In general, infants acquire their microbiome from their mother’s vagina (natural birth), skin (cesarean birth) and environment (including the breast milk) emphasizing the role of the NICU’s microbiome for the development of a healthy microbiome (Penders et al., 2006; Dominguez-Bello et al., 2010; Brooks et al., 2014). Babies treated in NICUs are often underweight, from low birth weight (<2500 g) to extremely low birth weight (<1000 g). They have congenital abnormalities, or undergone surgery, and are therefore susceptible to nosocomial infections (Stover et al., 2001; Urrea et al., 2003; Couto et al., 2007). As in other ICUs, also NICU patients often develop life-threatening infections. Potentially pathogenic bacteria are found in various locations, such as diaper scales, drawer handles, keyboards, sink counters, and door buttons (Hewitt et al., 2013). Epidemiological studies have shown that infective bacteria can spread particularly well via air (Adler et al., 2005), infant incubators (Singh et al., 2005; Touati et al., 2009), sink drains (Bonora et al., 2004), thermometers (Van den Berg et al., 2000), as well as soap dispensers (Buffet-Bataillon et al., 2009) and toys (Naesens et al., 2009). Brooks et al. (2014) found that tubing, surfaces, incubators, and hands are the most important reservoirs and sources for colonizing the premature babies. They also detected that bacteria which later colonize infants’ guts can initially be discovered in NICU environmental samples (Brooks et al., 2014). At genus level, typical bacteria on NICU surfaces include *Staphylococcus, Enterococcus, Acinetobacter, Bacteroides, Burkholderia, Clostridia, Pseudomonas* and *Streptococcus* (Hewitt et al., 2013; Brooks et al., 2014), which are all known to include opportunistic pathogens that potentially are of great risk for immunocompromised patients. However, most of the bacterial genera detected on NICU surfaces are harmless to humans. If and how these interact with patients and other bacteria is still not understood.

### Cleaning Procedures At ICUs

Cleaning practices at ICUs are an important part of preventing the spread of multidrug resistant organisms, such as MRSA and VRE, which are associated with HAIs, prolonged stays in hospitals, increased mortality rates and higher healthcare costs (Daxboeck et al., 2006). The cleaning procedures in ICUs are strict, though the practices may vary between hospitals. Depending on the frequency and type of use, dedicated ICU staff and additionally outsourced cleaning personnel are responsible for cleaning hospital interior fittings thoroughly daily, weekly, monthly, or yearly. As one example, the hygiene and cleaning protocol of the ICU, Department of Internal Medicine Graz, Medical University of Graz, is shortly mentioned (listed frequencies are minimum demand): E.g., floor is cleaned daily, toilets are cleaned daily (staff toilet) or twice (visitor toilet), shower heads are cleaned once a week, waste is evacuated as necessary and garbage bins are cleaned daily; windowsills, racks, sinks and showers are cleaned daily; laundry is washed daily, vacuum cleaning is done weekly, umbrella holders are cleansed monthly, and telephones and shutters yearly. Exposed surfaces with direct human contact, such as door handles and sinks are cleaned at least daily with cleaning detergents and surface disinfectants. In case of contamination of highly infectious material, including certain viruses and bacteria such as Norovirus and *Clostridium difficile*, a detailed procedure for hand and surface contamination is given: the hands have to be decontaminated with a specific disinfectant detergents under a specific exposure time, depending on which pathogen has caused the epidemic (Cleaning and disinfection protocol, guideline 2000.3116, 7.4.2014. ICU, Department of Internal Medicine, Medical University of Graz).

Despite precise protocols and appropriate disinfectants, statistical analyses of data from hospitals have revealed that fatal infections are increasing with more efficient cleaning practices, suggesting that current procedures are inadequate to protect the susceptible patients from serious, life-threatening infections (Arnold, 2014). Efficient cleaning practices are known to decrease, but not eradicate the multidrug resistant organisms living on hospital surfaces (Dancer, 2008). Consequently, new cleaning technologies are being developed. These new methods include for example technologies that are both microbiologically effective and safe to use, such as hydrogen peroxide vapor, and UV light decontamination for terminal cleaning, as well as ultra-microfibers associated with a copper-based biocide (Blazejewski et al., 2011). Hydrogen peroxide vapor and UV light can reduce the amount of bacterial cells by at least four orders of magnitude, leading to far smaller risks for patients to acquire any multidrug-resistant bacterial infection (Boyce, 2016 and references therein). These cleaning methods are particularly effective with uneven surfaces and textures that are difficult to access with other methods (Blazejewski et al., 2011). Additionally, bacterial contamination and growth can be reduced by selecting antimicrobial material, such as copper, that can reduce bacterial burden and the possibility for patients to acquire HAI (Schmidt et al., 2015).

Other important factors for preventing infections in ICUs, beside strict cleaning protocols, are monitoring of microbial colonization and educational interventions of the cleaning procedures and results (Goodman et al., 2008; Carling, 2013). The Centers for Disease Control and Prevention (CDC) published guidelines for monitoring programs for health care workers to improve the environmental hygiene in hospitals, and to provide instant feedback and a possibility to improve the current procedures. These monitoring methods include direct observation of staff performance and protocol compliance, quantitative microbial detection by swab and agar slide cultures, fluorescent markers to identify the frequently touched surfaces, as well as adenosine triphosphate (ATP) bioluminescence for detecting both microbial and non-microbial ATP present in monitored surfaces (Guh and Carling, 2015).

### Summary And Outlook

Research has already shown that objective monitoring can significantly reduce the contamination of surfaces near patients, and can point out the weaknesses of current protocols (Goodman et al., 2008; Carling, 2013). Monitoring projects have shown that flat surfaces and textiles are easier to keep at the required cleanliness level, whereas more complex surface types, including doorknobs, handles and other irregular surfaces, including electronic equipment are more often cleaned with unsatisfactory quality (Goodman et al., 2008). Time pressure and lack of adequate instructions may also play a role when the set cleaning standards are not met (Goodman et al., 2008). For example, the 2010 CDC tool kit “Options for Evaluating Environmental Cleaning” offers specific instructions on how to implement monitoring and intervention programs (Carling, 2013). When HAIs are reduced in number via these infection controls and prevention programs, also substantial economic benefit can be achieved (Raschka et al., 2013).

Recently, a new and completely different perspective in defeating hospital pathogens has emerged: the interest has shifted from pathogenic bacteria toward the whole microbial communities thriving on different surfaces in hospitals and ICUs, and to a more microbial ecological perspective on how the microbes interact with their environment and other species (Arnold, 2014). It has been shown that a higher microbial diversity can prevent pathogenic infections (van Elsas et al., 2012; Pham and Lawley, 2014), and the idea of supporting the beneficial hospital microbiome by increasing the microbial diversity has raised great interest (Hewitt et al., 2013; Berg et al., 2015). However, the interaction between pathogenic bacteria, opportunistic strains, and harmful and beneficial microbes in ICUs, as well as in hospitals in general, are not yet understood and more research is still needed.

## Operating Rooms

### Modern Operating Rooms Structure and Air Quality Monitoring

Operating rooms (ORs) are important hospital wards where most surgical procedures are performed. These areas are subjected to strict cleaning procedures such as sterilization, disinfection and removal of contaminants (e.g., dust and organic waste). Cleaning and maintenance schedules are implemented for each OR according to the surgical procedures performed. All ORs should be cleaned at the beginning of the day, between each surgical procedure, and at the end of the day, followed by a weekly or each second week total clean-up of the entire OR including walls, floor and ventilation system. In addition, guidelines propose the daily exposure to UV radiation (Rutala et al., 2008; Lives, 2009; Gupta et al., 2015).

Operating rooms are part of operating theater complexes and these complexes are architecturally divided into four different zones based on the level of cleanliness with the bacterial burden decreasing from the outer to the inner zones. These zones are maintained by a differential decreasing positive pressure to prevent unfiltered air flow toward the inside of the ORs (Spagnolo et al., 2013; Külpmann et al., 2016). The four zones can be divided as follows: (a) a protective area that includes the changing rooms for all the medical personnel, administrative staff rooms, pre and post-operative rooms and the sterile and non-sterile stores; (b) a clean area that connects the protective area to the aseptic zone; (c) the aseptic zone which includes the ORs; (d) and the disposal area for each OR (Harsoor and Bhaskar, 2007).

Modern ORs are equipped with HVAC (Heating, Ventilation and Air Conditioning) systems to control environmental factors, namely temperature, relative humidity and air flow. The ventilation systems (e.g., with vertical flow, horizontal flow, or exponential laminar flow) are equipped with different filters according to the surgical procedures performed. Most ORs have a conventional ventilation system with filters that have an efficiency of 80–95% in removing particles ≥5 μm (Dharan and Pittet, 2002). In ORs used for orthopedic and other implant surgeries, the air is filtered through HEPA filters. These filters have an effectiveness of 99.97% in eliminating airborne particles of 0.3 μm size and above (Dharan and Pittet, 2002; Sehulster et al., 2003; Lives, 2009; Spagnolo et al., 2013).

Monitoring the air quality is recommended for each OR and is often checked by particle count, a method derived from industrial cleanroom standards. This method has been proposed to determine both the effectiveness of the filters in the ventilation system as well as to establish the level of biological contamination (Pasquarella et al., 2000; Gupta et al., 2015).

Many studies have argued that the results of the particle count method do not correlate with the bacterial count results (Landrin et al., 2005; Scaltriti et al., 2007; Cristina et al., 2012). Only two studies have shown that there is a correlation between the number of airborne particles and the number of CFUs. The presence of particles >5 μm size indicate microbiological contamination in the aerosol (Seal and Clark, 1990; Stocks et al., 2010).

To date, there is no international standard of allowed airborne microbial contamination in ORs. Most countries have their own standards: for example, in France the microbiological limits are between 5 and 20 CFU/m^3^, which are lower than the limits of the United Kingdom (35 CFU/m^3^) and Switzerland (25 CFU/m^3^) (Landrin et al., 2005; Cristina et al., 2012). However, facing the increasing use of particle count over microbiological sampling, many countries have established their standards in accordance with the International Standards Organization (ISO) 14644 – Cleanrooms and associated controlled environments^1^. It is proposed that ORs should meet the requirements of a cleanroom of ISO 6 or 7 (explanations see also section on cleanrooms). In contrast, in the ORs equipped with HEPA filters, the levels of an ISO 5 class should be reached (Scaltriti et al., 2007; Chauveaux, 2015).

Active microbial monitoring has been used in most studies as the main method to determine the air cleanliness (Edmiston et al., 2005; Landrin et al., 2005; Wan et al., 2011; Cristina et al., 2012; Birgand et al., 2015b). This method uses an air sampler to collect a known volume of air which is then blown on agar plates for cultivation-based analyses (Napoli et al., 2012).

Besides this method, Friberg et al. (1999) have shown that in ORs with laminar air flow the CFU counts on sedimentation plates is a more relevant indicator of bacterial contamination, with CFU levels not exceeding 350 CFU/m^2^/h (Friberg et al., 1999). In addition to the particle counter and microbial monitoring, other methods (e.g., ATP test, fluorescent particle counter) have been implemented to determine the microbiological contamination of the air and surfaces in the ORs. Griffith et al. (2000) proposed the use of ATP test together with bacterial culture to identify the contaminated surfaces in ORs, while Dai et al. (2015) suggested the use of fluorescent particle counter for real-time measurements of microbes present on aerosol particles (Griffith et al., 2000; Dai et al., 2015).

### Surgical Site Infections: Factors, Sources And Prevention

In OR environments, the presence of microorganisms is closely linked to increased incidence of acquired surgical site infections (SSIs). About 14–20% of all hospital acquired infections are SSIs, leading to an increase in morbidity and mortality, along with rising costs to the healthcare system due to an extended stay in the hospital (Birgand et al., 2015a). Most of the microbes causing SSIs have an endogenous source, the patient’s microflora. Occasionally, microorganisms acquired from an exogenous source, such as the ORs environment or health care personnel, can be the cause of the development of SSIs (Mangram et al., 1999; Spagnolo et al., 2013).

The factors that may lead to SSIs development are multifarious and can be divided into 3 main categories: (i) patient-related characteristics (e.g., age, obesity, diabetes mellitus and other diseases); (ii) characteristics of surgical procedures (e.g., duration of the operation, type of procedure, surgeon skills, hypothermia control, antibiotic therapy, surgical personnel behavior and equipment) and (iii) the OR environment (Mangram et al., 1999; Cristina et al., 2012; Spagnolo et al., 2013).

In most studies, the relation between these factors and the development of SSIs has been explored mainly by determining the number of particles in the OR under different conditions. The number of airborne particles varies during a surgical procedure being higher at the beginning due to patient installation and surgical bed preparation, and an increased movement of the medical personnel (Knobben et al., 2006).

Additionally, the surgical personnel and patients release skin particles (especially when the skin is dry), respiratory aerosols, dust particles and textile fibers containing viable microorganisms in the OR environment, therefore increasing the overall count of airborne particles (Dineen and Drusin, 1973; Mangram et al., 1999). Moreover, Cristina et al. (2012) have shown that the use of certain instruments (e.g., ultrasonic scalpel, laser tissue coagulation), which produce surgical smoke, increases the number of particulates in the OR air during surgical procedures, but the increasing number of particulates was not correlated with the microbial load.

Besides the presence of surgical personnel, their behavior can also lead to an increased number of microbiological particles. Several studies have shown that the number of persons present during a surgical procedure influences the number of airborne particles to a big extent, their movement leads to resuspension of any dust particle settled and the door opening rates cause an increase in the number of bacteria that can enter the ORs (Scaltriti et al., 2007; Lynch et al., 2009; Wan et al., 2011). To lower the particles shed by the health care personnel and to decrease the incidence of SSIs, different guidelines suggest the use of alcohol-based hand rubs, double gloves, face masks, hoods for covering the hair as well as the use of disposable impermeable garments made of non-woven particles during surgical procedures (Sehulster et al., 2003; Howard and Hanssen, 2007; Humphreys, 2009; Lives, 2009; Salassa and Swiontkowski, 2014). In some studies, the incidence of SSIs increased when the health care personnel wore the suits and shoes or used mobile devices both in and out of the ORs (Amirfeyz et al., 2007; Hee et al., 2014; Venkatesan et al., 2015).

Up to 30% of all SSIs are known to be caused by *Staphylococcus aureus*, especially the methicillin-resistant strains (Anderson et al., 2007). *S. aureus* is one of the most commonly isolated microorganisms from the ORs environment and a typical skin-associated microbe, indicating that ORs are dominated by human associated microbiota (Shin et al., 2015).

In two different studies the number of *S. aureus* has been investigated in different zones of the ORs. The number was increased in the critical zone (in close proximity of the patient) in comparison with the intermediate and peripheral zone (Edmiston et al., 2005; Genet et al., 2011).

Besides *Staphylococcus* ssp., other microorganisms have been isolated from ORs such as: *Enterobacter* spp., *Micrococcus* spp., *Acinetobacter* spp., *Brevibacterium* spp., *Pseudomonas* spp., *Klebsiella* spp., *Bacillus* spp., and *Escherichia coli* (Edmiston et al., 2005; Wan et al., 2011; Al Laham, 2012; Venkatesan et al., 2015; Verde et al., 2015).

Commonly, the microbiota associated with SSIs are investigated by culture-dependent methods and include well known opportunistic pathogens (e.g., *S. aureus*, *Enterococcuss* spp., *Pseudomonas* spp., and *Escherichia coli*). However, a study performed by Wolcott et al. (2009) shows that the vast majority of the microorganisms linked to SSIs is unidentifiable using standard culture methods (Wolcott et al., 2009) and consists mostly of anaerobes (the majority belonging to the genus *Bacteroides*).

### The Microbiome Of Operating Rooms

Knowing that only a small fraction (around 1%) of the microbial diversity can be cultured and described (Amann et al., 1995), the usage of molecular methods arises as a prerequisite not only for identifying the microorganisms present in the ORs environment, but also for uncovering the mechanisms of their dispersal and exploring the sources of microbiological contamination.

To date, only one study has explored the entire microbiome of an OR by using molecular techniques (Shin et al., 2015). Shin et al. (2015) performed next generation sequencing of the microbial communities present in three OR environments (found in two different hospitals), and proved that the OR dust contained a microbial community similar to the one found on human skin (dominated by *Staphylococcus* and *Corynebacterium*). Moreover, *Staphylococcus* strains have been isolated from the dust present on ORs mobile surgery lamps, pointing out a high infection risk associated with the formation of microbial plumes. Overall, the study showed that the microbial communities present in all three ORs were similar, and that the bacteria present belonged to the phyla Proteobacteria, Firmicutes, Bacteroidetes, Actinobacteria, and Cyanobacteria (Shin et al., 2015).

More studies on the microbiome of the OR environment are needed to identify the main sources of microbial contamination, to understand how these microbes thrive in these controlled environments and how they are transmitted from humans to surfaces and *vice versa*. This would help to optimize stringent maintenance and cleaning procedures and to lower the microbial burden. Furthermore, health care personnel should be instructed on how to perform safer surgeries and how to minimize the microbial shedding during surgical procedures. The recommendations of WHO and CDC guidelines (Sehulster et al., 2003; Lives, 2009) should be applied in each OR to prevent SSIs and avoid unwanted expanse for both the patient and health care facilities.

## Cleanrooms

### Cleanrooms: Definition, Architectures and Classes

Cleanrooms are facilities used for ensuring quality and safety of many production processes. They are either mainly particulate-controlled (e.g., microelectronics, semi-conductor industry), or additionally biocontamination-controlled in case of food technology, pharmaceutical industry, medical processes (e.g., biosafety labs), aeronautics and many other application areas (Whyte, 2010).

The idea to use a biocontamination-controlled, clean environment to increase hygiene standards was first implemented by the two physicians Semmelweis and Lister in the 19th century. They realized the presence of an “invisible threat,” which we nowadays have identified as the presence of (opportunistic) pathogenic microorganisms or viruses. By their developed countermeasures they were able to significantly decrease mortality rates in hospitals (Semmelweis, 1988). However, it was Willis Whitfield who created the basis of the modern cleanroom in 1960 and solved the problem of contaminating particles and unpredictable airflows by the application of a constant highly filtered air flow to flush out air impurities (Whitfield, 1964). A “clean” production process results in a product, which is free of contaminants of concern. Such contaminants can be microorganisms themselves and their remnants, biomolecules in general, as well as any (inorganic) particulate matter that could affect the production process and the quality of the end product. Nowadays any outdoor air entering the cleanroom is filtered and air inside the facility is constantly recirculated through HEPA (high-efficiency particulate air) and/or ULPA (ultra-low particulate air) filters to prevent contaminants to enter the cleanroom or settle on its surfaces. In addition, most cleanrooms are operated at higher pressures than their outside environment to prevent inadvertent airflows into cleaner areas^2^.

The installation of a clean production line requires proper planning prior to the operation itself, including consideration of specific requirements of the product (Whyte, 2001). Specific decisions have to be taken with respect to operation (i.e., exchange of materials (products) and personnel), maintenance and monitoring (i.e., measurements of air conditions, particles, flow dynamics, acoustics, electrostatics, electromagnetics, contaminating sources, risk and hazard assessments, concepts of air flow facilities, laminar flow cabinets, filter fan units), calculations of energy and media consumptions, as well as hygiene protocols and evaluations (i.e., disinfection, decontamination).

A cleanroom class is defined by its amount of particles of a certain size according to the ISO classification criteria (see also above). Hence, a cleanroom of ISO Class 6 is for instance allowed to contain 10^6^ particles equal to and larger than 0.1 μm in size per m^3^ of air. This number is then decreasing by 1 log per ISO category resulting in 10^5^ for ISO 5, 10^4^ (ISO 4), 10^3^ (ISO 3), 10^2^ (ISO 2), and 10 particles for ISO 1, which represents the cleanest level. In case even higher cleanliness is required, so-called insulators can be installed inside a cleanroom environment. Cleanrooms of ISO classification 7–8 represent the most common and appropriate levels of cleanliness for many different production lines. Here, classification is based on 0.5 μm- sized and larger particles with limits at 3.5 × 10^5^ for ISO 7 and 3.5 × 10^6^ for ISO 8 per m^3^ air, whereas ISO Class 9 (3.5 × 10^7^ particles) corresponds already to the particle concentration observed in uncontrolled areas. Besides the presence of particles, cleanrooms are controlled with respect to temperature and humidity (HVAC systems; heating, ventilating and air conditioning), the kind and quality of gaseous substances, the light source, electrostatics and electromagnetics (Whyte, 1999; Hortig, 2002).

### Technologies for a Clean Production

Cleanrooms are often arranged in a sequential manner to guarantee desired conditions on each level. For this purpose, cleaner areas are only accessible after passing other cleanrooms of higher ISO classes in decreasing manner. Passages between different ISO classes and into cleanrooms are often sealed by airlocks or sluice systems, which sometimes include additional air showers and tacky mats. These systems intend to remove dust, soil, skin flakes and many other contaminating particles associated with a person or item. Work processes, as well as people behavior and interaction with respective products are strictly predefined to avoid needless spreading of particles. Hence, people in general are advised to perform their duties with slow body movements inside a cleanroom environment. In addition, the staff is equipped with special cleanroom garment that has to be donned in a specific area in a pre-defined order and often includes an overall, pants, bonnet, mustache cover, glasses, gloves, shoe covers, boots, and hoods. Previous studies have shown that dispersion rates of microbe carrying particles (MCPs; ≥0.5 μm) were substantially reduced from 2.1 × 10^6^ to 1 × 10^6^ per minute, when staff wore cleanroom garment compared to normal indoor clothing (Whyte and Hejab, 2007), emphasizing the effectiveness of such control measures.

Since cleanrooms can harbor entire production lines, these rooms are modular and scalable up to enormous sizes. Depending on the mode of use, cleanrooms can be equipped with diverse machines and furniture. Regardless of its special requirements, installed devices have in common that they should generate minimal air contaminations and are easy to clean. Hence, materials from natural fibers are often excluded from devices used in cleanrooms (Whyte, 2010).

Microbial decontamination actions are performed regularly but without leaving any residues behind. Standard cleaning reagents include alcohols (e.g., 70% (v/v) isopropanol), hydrogen peroxide (e.g., Klercide-CR) and alkaline cleaning reagents (e.g., Kleenol 30 or Jaminal Plus), and could be supplemented with, e.g., UV light, γ – irradiation and vapor-phase H_2_O_2_ treatments. Cleaning schedules can be rather elaborative including extensive repetitions of vacuuming and mopping as well as other cleaning protocols. As a result, microbial abundance is often intensively reduced compared to uncontrolled adjoining facilities. However, harsh environmental conditions and selective pressures in the cleanrooms also result in a microbial shift toward survival specialists like bacterial spore formers or archaea (Mahnert et al., 2015b).

### Microbial Monitoring in Cleanrooms

Microbial monitoring in biocontamination-controlled clean-rooms is often executed according to standard, cultivation dependent approaches based on the usage of contact plates (nutrient agar plates), witness plates (if specific surfaces are too sensitive to be sampled) or air sampling directly onto nutrient agar plates. Besides pharmaceutical cleanrooms, also industrial cleanrooms are sometimes required to operate under biocontamination control. Examples are spacecraft assembly cleanrooms that house mission vehicles, intended to land on extraterrestrial areas of elevated risk for contamination with Earth-borne microbes. Such missions are subject to strict planetary protection regulations (Kminek and Rummel, 2015).

First studies that examined the microbial contamination of such industrial cleanrooms were conducted in the 1960s (Nicholson et al., 2009), especially in preparation for the Viking mission to planet Mars (Puleo et al., 1977), starting with the microbial characterization of laminar flow cleanrooms (Powers, 1965). A first report on a comprehensive analysis of a horizontal laminar flow, three conventional industrial cleanrooms, and three open factory areas for the presence of microbial contaminants using witness plates was published by Favero et al. (1966). It was found that the number of CFUs was reduced along with the reduction of particles in samples from the air and surfaces and reached a plateau after several weeks of exposure. Microbial contaminations (mainly vegetative microorganisms of human origin like *Staphylococcus*, *Micrococcus*, *Corynebacterium*, *Brevibacterium*) could be clearly associated with the density and activity of personnel in the cleanroom (Favero et al., 1966). In the 60’s, general microbial levels on flat surfaces were evaluated using Rodac (Replicate Organism Detection and Counting) plates. These plates contained Trypticase Soy Agar (TSA) and were, after sampling, incubated at 32°C for 43 h (Vesley et al., 1966). Similar procedures are still used today. Later on, industrial cleanrooms were brought into a broader perspective after comparing their microbial contamination type and levels with those found in hospital ORs. The hospital environment harbored at least 1 log higher microbial abundances (based on colony forming units) than the investigated cleanrooms (Favero et al., 1968).

In the case of bioaerosol characteristics, Li and Hou (2003) observed only weak relationships among different cleanroom class levels in hospitals and air particle concentrations. The index of microbial air contamination (IMA) was proposed as a reliable tool for monitoring surface contamination by settling of microbes from the air and was tested in environments like hospitals, food industries, art galleries, aboard the MIR space station and in open air (Pasquarella et al., 2000).

Several authors discussed the effectivity of microbiological methods and analytical tools to assess the risk of typical microbial contaminants, such as *Staphylococcus*, *Microbacterium* and *Bacillus* (Wu and Liu, 2007) during pharmaceutical production (Whyte and Eaton, 2004a,b) or in aseptic processing cleanrooms (Hussong and Madsen, 2004). Thomas et al. concluded that the aseptic techniques applied by the personnel were more critical in avoiding contamination, than the general level of cleanliness of the environment (e.g., a cleanroom) for compounding drugs (Thomas et al., 2005).

Nevertheless, besides modeling the spreading of contaminants, risk assessments, improving sampling strategies from air and surfaces in various cleanroom settings, most studies that tried to expand applied methods beyond routine microbial monitoring were conducted in spacecraft assembly cleanroom settings due to planetary protection requirements (Nicholson et al., 2009; Kminek and Rummel, 2015). For planetary protection purposes, the profound knowledge and understanding of the cleanroom and spacecraft associated microorganisms is an important prerequisite for mission success. Besides standard assays based on cultivation of aerobic mesophilic and heat-shock resistant microorganisms, more sophisticated methods have been established. These included for instance the cultivation of microbial contaminants on anoxic TSA, resulting in a collection of more than 100 strains of facultative (*Cellulomonas, Paenibacillus, Staphylococcus, Arsenicicoccus, Dermabacter, Pseudomonas, Stenotrophomonas, Corynebacterium, Enterococcus*) and obligate anaerobes like *Clostridium* and *Propionibacterium* (Stieglmeier et al., 2009; Probst et al., 2010). Isolated bacteria from several spacecraft assembly cleanrooms were extensively tested for their resistance against numerous environmental stresses like desiccation, UV-C irradiation, γ-radiation, 5% (v/v) hydrogen peroxide, temperature extremes from 4 to 65°C up to a heat shock of 80°C, pH 3 and 11, and hypersalinity of 25% NaCl (w/v), in order to understand their potential capacity to survive space flight or under extraterrestrial conditions. Besides extremotolerant *Alphaproteobacteria*, *Betaproteobacteria*, *Gammaproteobacteria* (*Acinetobacter radioresistens*), *Actinobacteria* and fungi (*Aureobasidium*), highly tolerant spore forming isolates were found, including numerous bacilli, *Geobacillus* (thermophilic), *Paenibacillus* (obligate anaerobes), and other species that revealed halotolerant and alkalo-tolerant characteristics (La Duc et al., 2003a, 2007).

The application of diverse cultivation strategies and regular monitoring and isolation of microbes from spacecraft assembly cleanrooms resulted in a rich culture collection of extremotolerant microorganisms from confined built environments that is now open to the scientific community at the German Collection of Microorganisms and Cell Cultures DSMZ (Moissl-Eichinger et al., 2012, 2013) or through the U.S. Department of Agriculture’s Agricultural Research Service Culture Collection (Venkateswaran et al., 2014b).

### Targeting Microbial Communities Of Cleanrooms With Molecular Cultivation-Independent Technologies

However, beside cultivation based methods, several studies conducted in spacecraft assembly cleanrooms included also (molecular) cultivation independent assays to target microbial diversity and abundance in NASA (National Aeronautics and Space Administration) and ESA (European Space Agency) affiliated spacecraft assembly cleanrooms. La Duc and coworkers used molecular methods in 2003 in addition to culture-based methods to characterize microbial diversity of a cleanroom encapsulation facility and the collocated Mars Odyssey spacecraft. Predominant species in clone libraries included *Variovorax*, *Ralstonia* and *Aquaspirillum*. The application of various biomarkers such as ATP, LPS (lipopolysaccharides), and DNA to assess contamination of spacecraft and associated environments were reviewed by La Duc et al. (2004) including even samples from the International Space Station (ISS). In 2009, DNA microarrays (PhyloChip) were added and compared in-depth to standard cloning methods in a study covering cleanrooms before and after spacecraft assembly at Lockheed Martin Aeronautics Multiple Testing Facility (LMA-MTF), Kennedy Space Center Payload Hazard and Servicing Facility (KSC-PHSF), and the Jet Propulsion Laboratory Spacecraft Assembly Facility (JPL-SAF; La Duc et al., 2009). Three geographically distinct spacecraft-associated cleanrooms (Jet Propulsion Laboratory, Kennedy Space Flight Center, Johnson Space Center), including air samples, were analyzed in another study to determine if microbial populations are influenced by the surrounding environment or cleanroom maintenance. Only a small subset of microorganisms (e.g., *Acinetobacter*, *Deinococcus*, *Methylobacterium*, *Sphingomonas*, *Staphylococcus*, and *Streptococcus*) was common to all locations, whereas samples from Johnson Space Center featured the greatest diversity of bacteria, Kennedy Space Flight Center samples were characterized by a high presence of *Proteobacteria* and areas in the Jet Propulsion Laboratory assembly facility harbored mainly *Firmicutes*. The air of these spacecraft assembly facilities contained for instance *Massilia timonae*, *Agrobacterium tumefaciens* and *Agrobacterium sanguineum*, *Janthinobacterium lividum*, *Wautersia metallidurans*, *Acidovorax temperans*, *Deinococcus geothermalis*, *Delftia acidovorans*, *Gemmata obscuriglobus*, and *Methylobacterium fujisawaense* (Moissl et al., 2007). In addition to NASA operated spacecraft assembly cleanrooms, their European counterparts used by ESA were investigated for their microbial abundance and diversity as well (Stieglmeier et al., 2012). However, not only Bacteria could be associated to human-controlled environments but also signatures of Archaea (*Thaumarchaeota*, closely related to *Nitrososphaera gargensis*; and *Euryarchaeota* like halophilc and alkaliphilic *Halalkalicoccus*, and the methanogen *Methanosarcina*) were detected by molecular methods and could be visualized by FISH (fluorescence *in situ* hybridization; Moissl et al., 2008; Moissl-Eichinger, 2011).

Similarly like Bacteria, Archaea seem to be transferred by humans into cleanroom environments (Probst et al., 2013). Although they were found to be less (3 logs) abundant than bacteria (2.2 × 10^4^ archaeal cells per m^2^ cleanroom surface determined via quantitative PCR), they seem to be a constant microbial contaminant. Recently, an shotgun metagenomic approach using multiple displacement amplification (MDA) completed the picture of microbial life in a cleanroom by the detection of Eukaryotes (*Acanthamoeba* and fungi, e.g., *Leotiomyceta*, *Exophiala*, *Mycosphaerella*) and diverse viruses (Weinmaier et al., 2015).

New molecular methods like next generation sequencing nowadays allow not only a much better assessment of the total microbiome inside confined habitats like cleanrooms, but can additionally be enriched by different assays to target potential viable microbial communities. For instance the application of propidium monoazide (PMA), a chemical compound that masks DNA of dead cells from further downstream molecular analysis, revealed a remarkable proportion of dead cells (up to 99%) compared to other uncontrolled built environments (Vaishampayan et al., 2013; Mahnert et al., 2015b). The viable portion of the cleanroom environment included bacterial spore formers, such as *Ammoniphilus, Bacillus, Brevibacillus, Clostridium, Cohnella, Desulfosporosinus, Geobacillus, Paenibacillus, Planifilum, Sporosarcina, Terribacillus, Thermoactinomyces, Virgibacillus*) and Archaea (*Haloferax* and Candidatus *Nitrososphaera*; Vaishampayan et al., 2013; Mahnert et al., 2015b).

Moreover, viability assays using PMA were shown to increase the traceability of low abundant taxa of the rare viable biosphere (Mahnert et al., 2015b) and help to assess the entire complexity of microbiomes in confined environments which are dominated by DNA signatures of dead cells (Weinmaier et al., 2015). Hence, the importance to include differentiated methods targeting the total microbiome and that of viable or intact cells is of particular relevance in microbially controlled low biomass environments, to allow a less biased picture of the microbial DNA-based inventory.

The investigation of a whole cleanroom facility including adjoining facilities besides actual controlled cleanrooms highlighted the critical role of the gowning area. These areas are located in front of restricted clean zones, and were identified as the major location and source of microbial contaminant dispersal into cleanrooms (Moissl-Eichinger et al., 2015). Moreover, the authors of this study applied a broad spectrum of methods and compared standard cultivation techniques (TSA, R2A), adapted cultivation protocols for anaerobes (anoxic TSA), alkaliphiles (R2A at pH 10), halophiles, oligotrophes (RAVAN agar), methanogens (*Methanosarcina* medium) and various (molecular) cultivation-independent methods including 16S rRNA gene cloning, micro-array technology (PhyloChip) and next generation sequencing (454-pyrosequencing). Interestingly, against expectations, high throughput next generation sequencing technologies could not cover all cultivated microbes (Moissl-Eichinger et al., 2015). However, due to targeting 16S rRNA genes, this study missed the entire microbial complexity as accessible through broader or even untargeted approaches (Vaishampayan et al., 2013; Mahnert et al., 2015b). Hence all methods, even state-of-the-art, have their individual advantages, disadvantages and limitations. However, in combination they have the potential to lead to a more complete picture of microbes inside the extreme environment of the cleanroom (Moissl-Eichinger et al., 2015).

### Conclusion

In conclusion, from a microbial perspective, a cleanroom is an extreme environment, where strict maintenance and overall lack of nutrients complicate microbial growth. The human body serves as a continuous source of microbial contaminants, although also environmental sources (such as soil, dust particles and aerosol droplets) represent another common source of cleanroom microbes. Once transferred to the cleanroom environment, microbes adapt their metabolism (Weinmaier et al., 2015) to withstand harsh conditions, responding to starvation, by reduction of overall metabolic activity (dormancy) and spore formation. Hence, cleanroom maintenance selects especially for microbial adaptation and survival specialists – and thus enriches microbes posing a higher risk for planetary protection. For those purposes, cleanroom maintenance and the design of its infrastructure should be reconsidered and the necessity as well as impracticality of overall sterility in a cleanroom should be critically discussed in the future.

## ISS and Human Longterm Space Travel (Mars and Beyond)

### The International Space Station as a habitat

Another confined man-made habitat exists about 400 km above ground: The ISS, one of the biggest and most complex international scientific projects in history, is circling our planet in low Earth orbit. As joint venture of the five space agencies of USA (NASA), Europe (ESA), Russia (Roscosmos; Russian Federal Space Agency), Canada (CSA; Canadian Space Agency), and Japan (JAXA; Japanese Aerospace Exploration Agency), the ISS is organized in modules. The first module, namely the Russian Zarya module, was launched in 1998 and since 30th October 2000, the ISS has been constantly inhabited by humans. While the ISS kept growing by the addition of new modules over the years, also the crew size increased from initially three crew members to six international astronauts and cosmonauts wo are now routinely inhabiting the ISS. Naturally, the presence of humans also imposes the presence of their associated microorganisms in this confined habitat. Besides the arrival of new crew members roughly every 6 months and about one cargo transporter per month, delivering supplies and scientific equipment for experiments, the ISS is cut off from any other biological environment. Therefore, the ISS composes the most confined man-made and inhabited environment to date. In addition to its confinement, the ISS represents a very unusual microbial biotope. Higher radiation levels than on Earth, low nutrient levels due to reduced introduction of new material, constant temperature (~22°C), stable humidity (~60%) and microgravity characterize the ISS habitat and make it a unique and extreme-situated indoor environment (Coil et al., 2016).

### Microbial Safety Measures And Risk Factors

The microbiology on the ISS has been under surveillance since its first inhabitation. Standardized monitoring of surface and air samples onboard the ISS as well as more detailed post-flight investigations thereof have been conducted (Pierson, 2001; Castro et al., 2004; Alekhova et al., 2005, 2016; Novikova et al., 2006; Vesper et al., 2008; Satoh et al., 2011; Venkateswaran et al., 2014c; Checinska et al., 2015; Ichijo et al., 2016; Yamaguchi et al., 2016). Moreover, cleanliness of the ISS water supplies has been investigated (La Duc et al., 2003b; Bruce et al., 2005). The greater part of the first microbial investigations were mainly based on cultivation of bacteria and fungi on commercial high-nutrient media and under moderate conditions (Castro et al., 2004; Novikova et al., 2006; Van Houdt et al., 2012). Since Roscosmos could observe serious problems due to microbial contaminations during operation of the space station Mir, all involved space agencies agreed on preventive measures to protect spacecraft, cargo, and crew from harmful microorganisms (e.g., Novikova, 2004; Ott et al., 2014).

For example, the air regeneration system is equipped with HEPA or equivalent filters (POTOK 150MK in Russian modules) to remove airborne microorganisms and particles ≥0.3 μm. The acceptability limits for airborne bacteria and fungi were set to 10,000 and 100 CFUs/m^3^ of air, respectively. For surfaces the respective limits were defined with 10,000 CFUs/100 cm^2^ and 100 CFUs/100 cm^2^. The microbial limits for the ISS water supplies differs between the US and the Russian segments: US water must be free of coliforms, with a total heterotrophic content of less than 100 CFUs/100 mL, while the Russians allow heterotrophic bacteria up to 10,000 CFUs/100 mL (Pierson, 2001; Van Houdt et al., 2012).

In order to avoid higher levels of microbial contamination, a rigorous housekeeping program is in place that includes weekly cleaning, biweekly disinfection and standard monitoring of ISS air and surfaces for viable bacterial and fungal contaminants every 90 days. The used disinfection agents are either based on a quaternary ammonium compound which is supplied by the US or on the combination of a quaternary ammonium compound with hydrogen peroxide, which is supplied by the Russians (Directorate, 2000; Pierson, 2001; Castro et al., 2004; Novikova et al., 2006; Duane et al., 2011; Van Houdt et al., 2012). Monitoring of the microbial community onboard the ISS is highly important to evaluate material integrity of the spacecraft and to assess risk factors to the health of crew members. It is known that the human immune system is compromised under space conditions. For example, there is a significant decrease of lymphocytes and also the activity of innate and adaptive immune response is reduced compared to terrestrial controls (Sonnenfeld and Shearer, 2002; Aponte et al., 2006). Additionally, it has been shown that the virulence of most microorganisms is affected by microgravity. For some species virulence is enhanced in space conditions, such as *Salmonella typhimurium* (Wilson et al., 2007) and for some other species virulence is reduced, such as *Listeria monocytogenes* or *Enterococcus faecalis* (Hammond et al., 2013). It is also debated that the efficacy of antibiotics and other medications decreases under space conditions (Taylor, 2015).

Even the integrity of the spacecraft itself can be compromised by microorganisms. So-called technophilic microorganisms, in particular fungi, are able to corrode alloys and polymers used in spacecraft assembly (Alekhova et al., 2005). These technophilic microorganisms caused major problems on the former Russian space station Mir (Novikova et al., 2001; Novikova, 2004).

### The International Space Station Microbiome And Its Origin

The main fungal genera detected onboard the ISS by cultivation were *Aspergillus* and *Penicillium* (Alekhova et al., 2005; Novikova et al., 2006; Venkateswaran et al., 2014c). These fungi were also found in higher abundance using different molecular approaches; however, Satoh et al., 2011 did not find any *Penicillium* in the Japanese Kibo module 1 year after its installation, but detected a predominance of skin-associated *Malassezia* (Satoh et al., 2011).

The main bacterial phyla detected onboard the ISS in air and on surfaces, by either cultivation or molecular methods, were Firmicutes and Actinobacteria. In cultivation-based assays, *Bacillus* and *Staphylococcus* species were the most detected Firmicutes, whereas signatures of *Staphylococcu*s utterly dominate the Firmicutes-affiliated signatures detected by molecular methods. The most probable reason for this observed discrepancy might be the disability of standard DNA isolation protocols to open spores adequately (Venkateswaran et al., 2014c).

This finding emphasizes that cultivation approaches – although generally not able to record the whole diversity of a given environment (also stated above) – are still necessary for regular monitoring procedures. However, the ability of modern culture-independent molecular methods to assess the total microbial diversity present in a given environment is a powerful tool which enables researchers to elucidate the microbial community structure within the ISS beyond the standard cultivation assays. Next generation sequencing is nowadays also facilitating the microbiome analysis of the ISS. For instance, vacuum cleaner dust and filter debris collected from HEPA filters within the US American part of the ISS were analyzed in detail (Venkateswaran et al., 2014c) and their microbial inventory was also compared to the microbial inventory from spacecraft assembly cleanrooms (Checinska et al., 2015). Overall, there are several current projects which aim to broaden the knowledge about the ISS microbiome, including NASA’s “Microbial Observatory” project (Venkateswaran et al., 2014a), JAXA’s “Microbe” experiment series (Satoh et al., 2011; Ott et al., 2014; Ichijo et al., 2016; Yamaguchi et al., 2016) and ESA’s ARBEX project (Moissl-Eichinger et al., 2016).

Almost all studies which investigated the ISS microbiome agree in one major aspect: the crew members act as the main source for the ISS microbial community, since most of the detected microorganisms are known to be human associated. The only studies which did not report a dominance of microorganisms of a presumable anthropogenic origin were studies conducted on the water supplies of the ISS, which is reasonable since these should normally not come in extensive physical contact with humans. Most of the organisms in the ISS water supplies were gram negative Proteobacteria, such as *Methylobacterium*, *Sphingomonas*, *Ralstonia* and *Pseudomonas* (La Duc et al., 2003b; Bruce et al., 2005).

Besides the human body, the other possible contamination source in this secluded habitat is the cargo delivered to the ISS including food, general equipment and material for scientific experiments. Cargo is always subjected to adapted cleaning procedures before upload and should be at least “visibly clean” before sent to the ISS (Pierson, 2001; Mord, 2009).

The crew on the ISS wears clothing, which does not impede the dispersal of microorganisms off the respiratory tract or skin and thus is certainly the major reason for the predominance of *Staphylococcus* (Firmicutes), *Corynebacterium* and *Propionibacterium* (Actinobacteria), which were also proven to be present in a viable status (Venkateswaran et al., 2014c).

### Conclusion And Outlook

Many human associated fungal and bacterial species are known to be opportunistic pathogens which are able to infect people with a (severely) compromised immune system. As mentioned above, the human immune system is proven to be compromised in space and the virulence of some (opportunistic) pathogens could even be enhanced under space-flight conditions. Additionally, if left uncontrolled in a confined environment where environmental strains are not present, which would normally outcompete human associated microorganisms under such conditions, human associated microorganisms can easily proliferate quickly and thereby pose a health hazard, as has been shown in artificial closed ecosystems on Earth (e.g., Sun et al., 2016). However, to date, there has been no serious infection reported on board the ISS, and the above mentioned CFU limits were exceeded only in a few cases in which appropriate countermeasures succeeded in a timely manner (Van Houdt et al., 2012).

Taking all the publicly available information into consideration, one can conclude that the preventive measures which are in place on board the ISS are currently sufficient to ensure the safety of crew and spacecraft from the microbiological perspective. Nevertheless, the longitudinal analysis of microbial community behavior under space conditions is necessary to deliver crucial knowledge to enable future long term space missions, as e.g., a flight to Mars and beyond. For such a long-term spaceflight, not only the maintenance of a healthy microbiome in the human body and the surrounding environment has to be considered, but also the safe production of food and recycling of water. Spaceflight simulations, such as MARS 300 and MARS 500, and microbial monitoring thereof (Project: MICHA, DLR Cologne) are extremely helpful in order to elucidate potential pitfalls during a flight to Mars and beyond. However, much more research in this area is needed to ensure the health and well-being of the crew during such missions.

Recent and current studies on the overall microbial communities onboard the ISS help to understand the influence of microorganisms on this special inhabited confined environment, as well as on other man-made environments on Earth (and *vice versa*). The overwhelming majority of detected microorganisms are, however, no threat toward human health or material but provide tremendous resources for human body function, sustainable waste remediation, recycling and purification of water and/or air supplies as well as nutrients for renewable food sources or even as a renewable food source themselves (e.g., Nitta, 1999; Pierson, 2001; Czupalla et al., 2005; Bekatorou et al., 2006; Habib et al., 2008). In addition, the presence of beneficial microorganisms within a closed environment can help to suppress the harmful microbes and can thereby promote human health. As discussed in Mahnert et al. (2015a), this could potentially be achieved by installing plants in such confined environments, which could support indoor air quality, mental health, provide a food source and support human’s health and well-being by providing a natural microbiome source (Mahnert et al., 2015a). However, more research needs to be done in this regard, also to ensure that no harmful microorganisms are introduced by such plants.

## Addendum: High-Security Laboratories

High-security laboratories are facilities developing customized technological solutions covering functional and security needs in natural scientific sectors. The purpose of such a laboratory is to reduce or eliminate exposure of laboratory staff and the outside environment to potentially hazardous agents. Different biosafety levels (BSL) for bio-containment are defined to work with dangerous biological agents in an enclosed laboratory facility. Biological safety levels are ranked from one (BSL-1) to the highest level four (BLS-4) where high security labs are categorized into BSL-3 and BSL-4 based on the agents or organisms on which the research or work is being conducted. High-security laboratories, in particular of level 4, are thus the most confined environments, where humans work. Although not much is known about the indigenous microbial diversity in such environments, for the sake of completeness, these environments shall be mentioned shortly in this review, pointing to a lack of knowledge in this regard. BSL-3 includes safety equipment and construction which are applicable to clinical, diagnostic, teaching, research, or production facilities in which work is done with dangerous agents causing serious and potentially lethal infections. *Mycobacterium tuberculosis*, St. Louis encephalitis virus, and *Coxiella burnetii* are representative of the microorganisms assigned to this level (Wilson and Chosewood, 2007). Primary hazards to personnel working with these agents relate to autoinoculation, ingestion, and exposure to infectious aerosols. At BSL-3, more emphasis is placed on primary and secondary barriers to protect personnel in contiguous areas, the community, and the environment from exposure to potentially infectious aerosols. Thus, more protective barriers are used in BSL-3 laboratories, including tightly closed wraparound protective suits made of special materials like DuPont^TM^Tyvek^®^^3^ and respirators if required. A high-security laboratory does also comprise self-closing double-doors access apart from general building passageways and the ventilation must supply ducted systems for directional airflows without recirculation. BSL-4 is defined for working with dangerous and exotic agents that pose a high individual risk of life-threatening disease, which may be transmitted via the aerosol route and for which there is no available vaccine or therapy. Agents with a close or identical antigenic relationship to BSL-4 agents should also be handled at this level. BSL-4 microorganisms are the Ebola virus, the Lassa virus, and any agent with unknown risks of pathogenicity and transmission. Thus, BSL-4 facilities provide the maximum protection and containment. In addition to the BSL-3 level, there are requirements for complete clothing change in a special lock and decontamination of all materials when entering and leaving the laboratory. A BSL-4 facility is generally located in a separate building or a totally isolated zone within a building with proper supply and exhaust ventilation systems, where high-efficiency particulate air (HEPA) filters exhaust the air, depending on the agents used (World Health Organization, 2005). Additionally, the laboratories also have their own air, electricity and water supply and multi-level security systems to prevent that pathogens reach the outside.

Besides biological agents, BSLs comprise safe work practices, specialized safety equipment (primary barriers) and facility design (secondary barriers), which are summarized in different reports (US Department of Health and Human Services, 2009; McLeod, 2010). In the United States, the Centers for Disease Control and Prevention (CDC) have specified all BSL levels (Richmond and McKinney, 1999), whereas in the European Union, the same biosafety levels are defined in directives. The most important EU regulations for biosafety laboratories are directive for biological agents at work (2000/54/EC), workplace safety (89/391/EC), contained use of microorganisms (98/81/EC), deliberate release into the environment (2001/18/EC), hazardous waste (94/31/EC), or directive on harmful organisms, plants, plant products and other objects (95/44/EC). The CDC and the National Institutes of Health (NIH) are our main sources for biological safety information for infectious agents. High security laboratories are characterized by strict hygienic guidelines comprising qualified employees, measures, and disinfection- and cleaning plans. Disinfectants, dosage and applications are defined within SOPs. The common decontamination strategies are hydrogen peroxide (H_2_O_2_), formaldehyde (CH_2_O) or chlorine dioxide (ClO_2_). A study (Beswick et al., 2011) reported that these methods were tested of their efficacy where only chlorine dioxide and formaldehyde showed a high disinfection efficacy.

In comparison to other indoor environments, biosafety laboratories are even more confined. Wearing of special clothes and protections prevents the humans from microbes as well as particles, which can be a carrier of microorganisms. Microbial communities of mentioned indoor habitats within this review are well analyzed by several next generation sequencing methods, but no research study about microbiome analysis in high security laboratories exists. It can be assumed that microorganisms of this extreme habitat adapt to low nutrient and dry conditions as well as strict hygienic guidelines (e.g., decontamination procedures or lock systems), as it was observed for microorganisms of other confined habitats. Further, monitoring of high-security labs is becoming more and more important to prevent outbreaks of disease and to maintain public faith. Generally, the awareness is low, but in cases of epidemic, e.g., Ebola or SARS, the interest increases. Thus, the World Health Organization (WHO) has recently witnessed a worldwide increase in the demand for biosafety guidance and support that culminated in 2005 with the adoption by the World Health Assembly of resolution WHA 58.29 on enhancement of laboratory biosafety (World Health Organization, 2005).

## Conclusion

**FIGURE 2:**
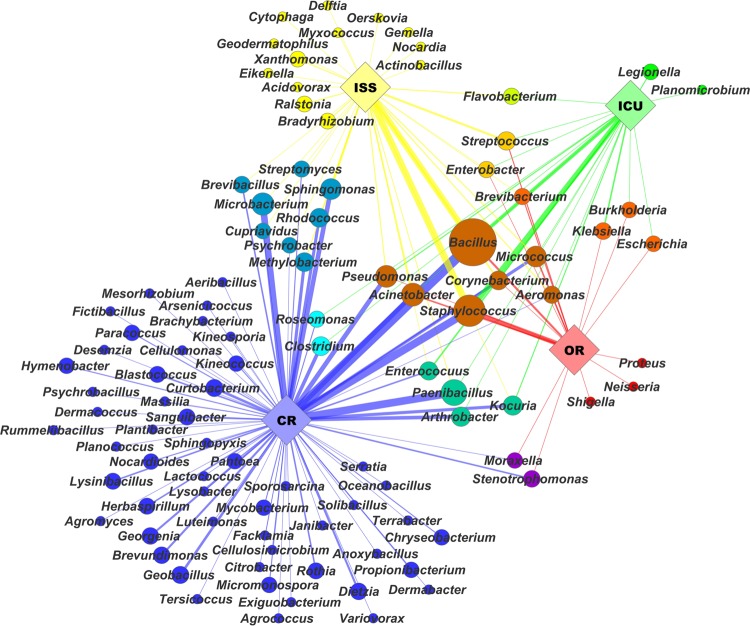
**The microbial network visualizes microbial profiles of selected confined habitats based on a range of isolates obtained from these environments.** The network was arranged with Cytoscape using a spring-embedded algorithm on eweights. Diversity of isolates on genus level was correlated with node size. Nodes and edges were colored by color mixtures of their respective environments: international space station (ISS) – yellow, intensive care units (ICU) – green, cleanrooms (CR) – blue, and operating rooms (OR) – red. Edge width and opacity was correlated to respective eweights, which were computed in QIIME (Caporaso et al., 2012).

Due to similar maintenance, architecture and type of confinement, the environments presented here harbor a very specific microbial core-community. ICUs, ORs, cleanrooms and even the ISS share a number of typical microbial inhabitants, as displayed in **Figure 2**. In particular *Bacillus* and human-associated microbial species are cultivated from all confined areas, reflecting the typical microbial community being composed of survival specialists (such as spore formers) and mainly representatives of the human microbiome, defining the human body as major source of microbial contamination.

The purposes for confinement are different. In the hospital area, the risk of infection is the major driving factor for confinement. Interestingly, higher efforts in cleaning (i.e., sterilization and bioburden reduction) do not necessarily decrease the risk for infections, in contrary: they were even correlated with a higher incidence of infections and presence of multi-resistant strains. Similar findings exist for cleanroom environments: the microbial inhabitants frequently showed higher resistances against physical and chemical stresses than their naturally occurring counterparts. All of these discussed habitats are extreme and pose stresses toward the internal microbiome, which entails a positive selection pressure for microbes which are adapted to such stresses and therefore promotes the development and establishment of survival strategies within these habitats.

Interestingly, the ISS seems to be a safe work space: despite allergic reactions (Venkateswaran et al., 2014c), so far no severe incidences of outbreaks have officially been reported. Certainly, although most confined, this is also an area where a higher number and diversity of microorganisms can be accepted, since neither persons, nor products are exposed to instantaneous risk.

Although cleanrooms are not living places for human beings, they have been subjected to comprehensive microbial analyses during the last years, using most sophisticated molecular and cultivation-based methods. While the overwhelming majority of cleanroom microorganisms appears to be dead, the survivors are specifically resistant and are considered possible contaminants, of e.g., spacecraft targeting potential extraterrestrial biotopes.

In all habitats considered in this review, the routes of microbial transmission are not clearly resolved yet, leading to uncertainty with respect to optimal maintenance and risk management. Based on our experience and the information summarized in this review, we argue, that hygiene and maintenance strategies need to be critically reviewed, and the role of beneficial microorganisms, that naturally suppress unwanted microorganisms, need to be reassessed. The most-likely healthy transfer of beneficial microorganisms through, e.g., pets or plants into patient rooms is currently restricted, due to uncontrollable risks. However, a controlled spreading of selected, beneficial microorganisms in certain settings could help tremendously to improve quality of living and human health and to reduce long-term risks emanating from multi-resistant microbial strains.

## Author Contributions

MM wrote chapter about ISS and organized manuscript writing. AM wrote chapter about clean rooms and prepared figures. KK wrote chapter about ICUs. MP wrote chapter about operating rooms. LO-W wrote chapter about biosafety laboratories. RK, AP, GG, and GB critically read the manuscript and provided discussion. CM-E wrote and composed the manuscript.

## Conflict of Interest Statement

The authors declare that the research was conducted in the absence of any commercial or financial relationships that could be construed as a potential conflict of interest.

## Footnotes

1 https://www.iso.org/obp/ui/#iso:std:iso:14644:-1:ed-2:v1:en

2 http://www.thomasnet.com/articles/automation-electronics/Cleanroom-Air-Flow-Principles

3 www.dupont.com

## References

[B1] Adler A. Gottesman G. Dolfin T. Arnon S. Regev R. Bauer S. (2005). Bacillus species sepsis in the neonatal intensive care unit. *J. Infec.* 51 390–395. 10.1016/j.jinf.2004.12.006 16321650

[B2] Agodi A. Barchitta M. Cipresso R. Giaquinta L. Romeo M. A. Denaro C. (2007). *Pseudomonas aeruginosa* carriage, colonization, and infection in ICU patients. *Intensive Care Med.* 33 1155–1161. 10.1007/s00134-007-0671-6 17503016

[B3] Al Laham N. A. (2012). Prevalence of bacterial contamination in general operating theaters in selected hospitals in the Gaza Strip, Palestine. *J. Infect. Public Health* 5 43–51. 10.1016/j.jiph.2011.10.006 22341842

[B4] Alekhova T. Aleksandrova A. Novozhilova T. Y. Lysak L. Zagustina N. Bezborodov A. (2005). Monitoring of microbial degraders in manned space stations. *Appl. Biochem. Microbiol.* 41 382–389. 10.1007/s10438-005-0065-x 16212041

[B5] Alekhova T. A. Zakharchuk L. M. Tatarinova N. Y. Kadnikov V. V. Mardanov A. V. Ravin N. V. (2016). Diversity of bacteria of the genus *Bacillus* on board of international space station. *Dokl. Biochem. Biophys.* 465 347–350. 10.1134/s1607672915060010 26728721

[B6] Amann R. I. Ludwig W. Schleifer K.-H. (1995). Phylogenetic identification and in situ detection of individual microbial cells without cultivation. *Microbiol. Rev.* 59 143–169. 7535888 10.1128/mr.59.1.143-169.1995 PMC239358

[B7] Amirfeyz R. Tasker A. Ali S. Bowker K. Blom A. (2007). Theatre shoes—a link in the common pathway of postoperative wound infection? *Ann. R. Coll. Surg. Engl.* 89 605–608. 10.1308/003588407X205440 18201476 PMC2121255

[B8] Anderson D. J. Sexton D. J. Kanafani Z. A. Auten G. Kaye K. S. (2007). Severe surgical site infection in community hospitals: epidemiology, key procedures, and the changing prevalence of methicillin-resistant *Staphylococcus aureus*. *Infect. Control Hosp. Epidemiol.* 28 1047–1053. 10.1086/520731 17932825

[B9] Anderson R. N. Smith B. L. (2005). Deaths: leading causes for 2002. *Natl. Vital Stat. Rep.* 53 1–89. 15786629

[B10] Aponte V. M. Finch D. S. Klaus D. M. (2006). Considerations for non-invasive in-flight monitoring of astronaut immune status with potential use of MEMS and NEMS devices. *Life Sci.* 79 1317–1333. 10.1016/j.lfs.2006.04.007 16757003

[B11] Arnold C. (2014). Rethinking sterile: the hospital microbiome. *Environ. Health Perspect.* 122 A182–A187. 10.1289/ehp.122-A182 24983914 PMC4080534

[B12] Beceiro A. Tomás M. Bou G. (2013). Antimicrobial resistance and virulence: a successful or deleterious association in the bacterial world? *Clin. Microbiol. Rev.* 26 185–230. 10.1128/CMR.00059-12 23554414 PMC3623377

[B13] Bekatorou A. Psarianos C. Koutinas A. A. (2006). Production of food grade yeasts. *Food Technol. Biotechnol.* 44 407–415.

[B14] Berg G. Mahnert A. Moissl-Eichinger C. (2015). Beneficial effects of plant-associated microbes on indoor microbiomes and human health? *Front. Microbiol* 5:15. 10.3389/fmicb.2014.00015 PMC3905206 24523719

[B15] Beswick A. J. Farrant J. Makison C. Gawn J. Frost G. Crook B. (2011). Comparison of multiple systems for laboratory whole room fumigation. *Appl. Biosaf.* 16 139–157. 10.1177/153567601101600303

[B16] Birgand G. Saliou P. Lucet J.-C. (2015a). Influence of staff behavior on infectious risk in operating rooms: what is the evidence? *Infect. Control Hosp. Epidemiol.* 36 93–106. 10.1017/ice.2014.9 25627767

[B17] Birgand G. Toupet G. Rukly S. Antoniotti G. Deschamps M.-N. Lepelletier D. (2015b). Air contamination for predicting wound contamination in clean surgery: a large multicenter study. *Am. J. Infect. Control* 43 516–521. 10.1016/j.ajic.2015.01.026 25752955

[B18] Blazejewski C. Guerry M.-J. Preau S. Durocher A. Nseir S. (2011). New methods to clean ICU rooms. *Infect. Disord. Drug Targets* 11 365–375. 21679145 10.2174/187152611796504818

[B19] Bonora M. G. Ligozzi M. De Fatima M. Bragagnolo L. Goglio A. Guazzotti G. C. (2004). Vancomycin-resistant *Enterococcus faecium* isolates causing hospital outbreaks in northern Italy belong to the multilocus sequence typing C1 lineage. *Microb. Drug Resist.* 10 114–123. 10.1089/1076629041310046 15256026

[B20] Boyce J. M. (2016). Modern technologies for improving cleaning and disinfection of environmental surfaces in hospitals. *Antimicrob. Resist. Infect. Control* 5:10 10.1186/s13756-016-0111-x PMC4827199 27069623

[B21] Brooks B. Firek B. A. Miller C. S. Sharon I. Thomas B. C. Baker R. (2014). Microbes in the neonatal intensive care unit resemble those found in the gut of premature infants. *Microbiome* 2:1 10.1186/2049-2618-2-1 PMC4392516 24468033

[B22] Bruce R. J. Ott C. M. Skuratov V. M. Pierson D. L. (2005). Microbial surveillance of potable water sources of the International Space Station. *SAE Trans.* 114 283–292.

[B23] Buffet-Bataillon S. Rabier V. Bétrémieux P. Beuchée A. Bauer M. Pladys P. (2009). Outbreak of *Serratia marcescens* in a neonatal intensive care unit: contaminated unmedicated liquid soap and risk factors. *J. Hosp. Infect.* 72 17–22. 10.1016/j.jhin.2009.01.010 19246120

[B24] Burmølle M. Webb J. S. Rao D. Hansen L. H. Sørensen S. J. Kjelleberg S. (2006). Enhanced biofilm formation and increased resistance to antimicrobial agents and bacterial invasion are caused by synergistic interactions in multispecies biofilms. *Appl. Environ. Microbiol.* 72 3916–3923. 10.1128/AEM.03022-05 16751497 PMC1489630

[B25] Califf K. Gonzalez A. Knight R. Caporaso J. G. (2014). The human microbiome: getting personal. *Microbe* 9 410–415.

[B26] Caporaso J. G. Lauber C. L. Walters W. A. Berg-Lyons D. Huntley J. Fierer N. (2012). Ultra-high-throughput microbial community analysis on the Illumina HiSeq and MiSeq platforms. *ISME J.* 6 1621–1624. 10.1038/ismej.2012.8 22402401 PMC3400413

[B27] Carling P. (2013). Methods for assessing the adequacy of practice and improving room disinfection. *Am. J. Infect. Control* 41 S20–S25. 10.1016/j.ajic.2013.01.003 23622743

[B28] Castro A. V. Thrasher N. A. Healy M. Ott M. C. Pierson L. D. (2004). Microbial characterization during the early habitation of the International Space Station. *Microb. Ecol.* 47 119–126. 10.1007/s00248-003-1030-y 14749908

[B29] Centers for Disease and Prevention (2002). *Summary of Notifiable Diseases-United States, 2000.* Morbidity and Mortality Weekly Report 49 Atlanta, GA: Centers for Disease and Prevention.

[B30] Centers for Disease and Prevention (2010). *Summary of Notifiable Diseases United States, 2008.* Mobidity and Mortality Weekly Report 57 Atlanta, GA: Centers for Disease and Prevention 54.

[B31] Chauveaux D. (2015). Preventing surgical-site infections: measures other than antibiotics. *Orthop. Traumatol. Surg. Res.* 101 (1 Suppl.) S77–S83. 10.1016/j.otsr.2014.07.028 25623269

[B32] Checinska A. Probst A. J. Vaishampayan P. White J. R. Kumar D. Stepanov V. G. (2015). Microbiomes of the dust particles collected from the International Space Station and spacecraft assembly facilities. *Microbiome* 3:50 10.1186/s40168-015-0116-3 PMC4624184 26502721

[B33] Coil D. A. Neches R. Y. Lang J. M. Brown W. E. Severance M. Cavalier D. D. (2016). Growth of 48 built environment bacterial isolates on board the International Space Station (ISS). *PeerJ* 4 e1842. 10.7717/peerj.1842 PMC4806633 27019789

[B34] Couto R. C. Carvalho E. A. Pedrosa T. M. Pedroso Ê. R. Neto M. C. Biscione F. M. (2007). A 10-year prospective surveillance of nosocomial infections in neonatal intensive care units. *Am. J. Infect. Control* 35 183–189. 10.1016/j.ajic.2006.06.013 17433942

[B35] Cristina M. L. Spagnolo A. M. Sartini M. Panatto D. Gasparini R. Orlando P. (2012). Can particulate air sampling predict microbial load in operating theatres for arthroplasty? *PLoS ONE* 7:e52809 10.1371/journal.pone.0052809 PMC3528722 23285189

[B36] Czupalla M. Horneck G. Blome H. J. (2005). The conceptual design of a hybrid life support system based on the evaluation and comparison of terrestrial testbeds. *Adv. Space Res.* 35 1609–1620. 10.1016/j.asr.2005.06.010 16175693

[B37] Dai C. Zhang Y. Ma X. Yin M. Zheng H. Gu X. (2015). Real-time measurements of airborne biologic particles using fluorescent particle counter to evaluate microbial contamination: results of a comparative study in an operating theater. *Am. J. Infect. Control* 43 78–81. 10.1016/j.ajic.2014.10.004 25564128

[B38] Dancer S. J. (2008). Importance of the environment in meticillin-resistant *Staphylococcus aureus* acquisition: the case for hospital cleaning. *Lancet Infect. Dis.* 8 101–113. 10.1016/S1473-3099(07)70241-4 17974481

[B39] Daniels C. Ramos J. L. (2009). Adaptive drug resistance mediated by root–nodulation–cell division efflux pumps. *Clin. Microbiol. Infect.* 15 32–36. 10.1111/j.1469-0691.2008.02693.x 19220351

[B40] Daxboeck F. Budic T. Assadian O. Reich M. Koller W. (2006). Economic burden associated with multi-resistant Gram-negative organisms compared with that for methicillin-resistant Staphylococcus aureus in a university teaching hospital. *J. Hosp. Infect.* 62 214–218. 10.1016/j.jhin.2005.07.009 16257092

[B41] Dharan S. Pittet D. (2002). Environmental controls in operating theatres. *J. Hosp. Infect.* 51 79–84. 10.1053/jhin.2002.1217 12090793

[B42] Dineen P. Drusin L. (1973). Epidemics of postoperative wound infections associated with hair carriers. *Lancet* 302 1157–1159. 10.1016/S0140-6736(73)92933-4 4127543

[B43] Directorate N. M. O. (2000). *International Space Station Integrated Medical Group (IMG) Medical Checklist. Mission Operations Directorate, Operations Division.* Houston, TX: National Aeronautics and Space Administration Available at: https://www.nasa.gov/centers/johnson/pdf/163533main_ISS_Med_CL.pdf

[B44] Dominguez-Bello M. G. Costello E. K. Contreras M. Magris M. Hidalgo G. Fierer N. (2010). Delivery mode shapes the acquisition and structure of the initial microbiota across multiple body habitats in newborns. *Proc. Natl. Acad. Sci. U.S.A.* 107 11971–11975. 10.1073/pnas.1002601107 20566857 PMC2900693

[B45] Donowitz L. G. Wenzel R. P. Hoyt J. W. (1982). High risk of hospital-acquired infection in the ICU patient. *Crit. Care Med.* 10 355–357. 10.1097/00003246-198206000-00001 7075228

[B46] Duane P. Rebekah B. Ott C. M. Victoria C. Satish M. (2011). “Microbiological Lessons Learned From the Space Shuttle,” in *Proceeding of the 41st International Conference on Environmental Systems* (Reston, VA: American Institute of Aeronautics and Astronautics).

[B47] Edmiston C. E. Seabrook G. R. Cambria R. A. Brown K. R. Lewis B. D. Sommers J. R. (2005). Molecular epidemiology of microbial contamination in the operating room environment: Is there a risk for infection? *Surgery* 138 573–582. 10.1016/j.surg.2005.06.045 16269284

[B48] Favero M. S. Puleo J. R. Marshall J. H. Oxborrow G. S. (1966). Comparative levels and types of microbial contamination detected in industrial clean rooms. *Appl. Microbiol.* 14 539–551. 5954479 10.1128/am.14.4.539-551.1966 PMC546777

[B49] Favero M. S. Puleo J. R. Marshall J. H. Oxborrow G. S. (1968). Comparison of microbial contamination levels among hospital operating rooms and industrial clean rooms. *Appl. Microbiol.* 16 480–486. 5649862 10.1128/am.16.3.480-486.1968 PMC547444

[B50] Fijan S. Turk S. S. (2012). Hospital textiles, are they a possible vehicle for healthcare-associated infections? *Int. J. Environ. Res. Public Health* 9 3330–3343. 10.3390/ijerph9093330 23202690 PMC3499872

[B51] Friberg B. Friberg S. Burman L. (1999). Inconsistent correlation between aerobic bacterial surface and air counts in operating rooms with ultra clean laminar air flows: proposal of a new bacteriological standard for surface contamination. *J. Hosp. Infect.* 42 287–293. 10.1053/jhin.1998.0542 10467542

[B52] Fux C. Costerton J. Stewart P. Stoodley P. (2005). Survival strategies of infectious biofilms. *Trends Microbiol.* 13 34–40. 10.1016/j.tim.2004.11.010 15639630

[B53] Gaci N. Borrel G. Tottey W. O’Toole P. W. Brugère J.-F. (2014). Archaea and the human gut: new beginning of an old story. *World J. Gastroenterol.* 20 16062–16078. 10.3748/wjg.v20.i43.16062 25473158 PMC4239492

[B54] Gastmeier P. Loui A. Stamm-Balderjahn S. Hansen S. Zuschneid I. Sohr D. (2007). Outbreaks in neonatal intensive care units—they are not like others. *Am. J. Infect. Control* 35 172–176. 10.1016/j.ajic.2006.07.007 17433940

[B55] Genet C. Kibru G. Tsegaye W. (2011). Indoor air bacterial load and antibiotic susceptibility pattern of isolates in operating rooms and surgical wards at Jimma University specialized hospital, Southwest Ethiopia. *Ethiop. J. Health Sci.* 21 9–18. 10.4314/ejhs.v21i1.69039 PMC3275854 22434981

[B56] Goodman E. R. Piatt R. Bass R. Onderdonk A. B. Yokoe D. S. Huang S. S. (2008). Impact of an environmental cleaning intervention on the presence of methicillin-resistant *Staphylococcus aureus* and vancomycin-resistant enterococci on surfaces in intensive care unit rooms. *Infect. Control Hosp. Epidemiol.* 29 593–599. 10.1086/588566 18624666 PMC2670228

[B57] Griffith C. J. Cooper R. A. Gilmore J. Davies C. Lewis M. (2000). An evaluation of hospital cleaning regimes and standards. *J. Hosp. Infect.* 45 19–28. 10.1053/jhin.1999.0717 10833340

[B58] Guh A. Carling P. (2015). *Options for Evaluating Environmental Cleaning.* Atlanta, GA: Centers for Disease Control and Prevention.

[B59] Gupta C. Vanathi M. Tandon R. (2015). Current concepts in operative room sterilisation. *Official Sci. J. Delhi Ophthalmol. Soc.* 25 190–194. 10.1089/sur.2014.097

[B60] Habib M. A. B. Parvin M. Huntington T. C. Hasan M. R. (2008). *A Review on Culture, Production and Use of Spirulina as Food for Humans and Feeds for Domestic Animals and Fish.* Rome: Food and Agriculture Organization of the United Nations.

[B61] Hammond T. G. Stodieck L. Birdsall H. H. Becker J. L. Koenig P. Hammond J. S. (2013). Effects of microgravity on the virulence of *Listeria monocytogenes*, *Enterococcus faecalis*, *Candida albicans*, and Methicillin-Resistant *Staphylococcus aureus*. *Astrobiology* 13 1081–1090. 10.1089/ast.2013.0986 24283929

[B62] Handorean A. Robertson C. E. Harris J. K. Frank D. Hull N. Kotter C. (2015). Microbial aerosol liberation from soiled textiles isolated during routine residuals handling in a modern health care setting. *Microbiome* 3 72 10.1186/s40168-015-0132-3 PMC4673858 26646166

[B63] Harsoor S. Bhaskar S. B. (2007). Designing an ideal operating room complex. *Indian J. Anaesth.* 51 193–199.

[B64] Hee H. I. Lee S. Chia S. N. Lu Q. S. Liew A. P. Q. Ng A. (2014). Bacterial contamination of surgical scrub suits worn outside the operating theatre: a randomised crossover study. *Anaesthesia* 69 816–825. 10.1111/anae.12633 24749987

[B65] Heeg P. Heizmann W. Mentzel H. (1994). Infections caused by Flavobacterium meningosepticum in patients in a neonatal intensive care unit. *Zentralbl. Hyg. Umweltmed.* 195 282–287. 8011057

[B66] Hewitt K. M. Mannino F. L. Gonzalez A. Chase J. H. Caporaso J. G. Knight R. (2013). Bacterial diversity in two neonatal intensive care units (NICUs). *PLoS ONE* 8:e54703 10.1371/journal.pone.0054703 PMC3553055 23372757

[B67] Hortig H.-P. (2002). “Systeme und konzepte der reinraumtechnik,” in *Reinraumtechnik* eds Gail L. Hortig H.-P. (Berlin: Springer) 1–18.

[B68] Howard J. L. Hanssen A. D. (2007). Principles of a clean operating room environment. *J. Arthroplasty* 22 6–11. 10.1016/j.arth.2007.05.013 17919585

[B69] Hppe P. Martinac I. (1998). Indoor climate and air quality review of current and future topics in the field of ISB study group 10. *Int. J. Biometeorol.* 42 1–7. 9780844 10.1007/s004840050075

[B70] Hu H. Johani K. Gosbell I. B. Jacombs A. S. W. Almatroudi A. Whiteley G. S. (2015). Intensive care unit environmental surfaces are contaminated by multidrug-resistant bacteria in biofilms: combined results of conventional culture, pyrosequencing, scanning electron microscopy, and confocal laser microscopy. *J. Hosp. Infect.* 91 35–44. 10.1016/j.jhin.2015.05.016 26187533

[B71] Huang S. S. Datta R. Platt R. (2006). Risk of acquiring antibiotic-resistant bacteria from prior room occupants. *Arch. Intern. Med.* 166 1945–1951. 10.1001/archinte.166.18.1945 17030826

[B72] Human Microbiome Project Consortium (2012). Structure, function and diversity of the healthy human microbiome. *Nature* 486 207–214. 10.1038/nature11234 22699609 PMC3564958

[B73] Humphreys H. (2009). Preventing surgical site infection. Where now? *J. Hosp. Infect.* 73 316–322. 10.1016/j.jhin.2009.03.028 19700219

[B74] Hussong D. Madsen R. E. (2004). Analysis of environmental microbiology data from cleanroom samples. *Pharm. Technol.* 28 10–15.

[B75] Ichijo T. Yamaguchi N. Tanigaki F. Shirakawa M. Nasu M. (2016). Four-year bacterial monitoring in the International Space Station–Japanese Experiment Module “Kibo” with culture-independent approach. *npj Microgravity* 2:16007 10.1038/npjmgrav.2016.7 PMC5515537 28725725

[B76] Kim K. H. Fekety R. Batts D. H. Brown D. Cudmore M. Silva J. (1981). Isolation of *Clostridium* difficile from the environment and contacts of patients with antibiotic-associated colitis. *J. Infect. Dis.* 143 42–50. 10.1093/infdis/143.1.42 7217711

[B77] Klevens R. M. Edwards J. R. Richards C. L. Jr. Horan T. C. Gaynes R. P. Pollock D. A. (2007). Estimating health care-associated infections and deaths in US hospitals, 2002. *Public Health Rep.* 122 160–166. 17357358 10.1177/003335490712200205 PMC1820440

[B78] Kminek G. Rummel J. D. (2015). COSPAR’s planetary protection policy. *Space Res. Today* 193 7–19.

[B79] Knobben B. Van Horn J. Van der Mei H. Busscher H. (2006). Evaluation of measures to decrease intra-operative bacterial contamination in orthopaedic implant surgery. *J. Hosp. Infect.* 62 174–180. 10.1016/j.jhin.2005.08.007 16343691

[B80] Kramer A. Schwebke I. Kampf G. (2006). How long do nosocomial pathogens persist on inanimate surfaces? A systematic review. *BMC Infect. Dis.* 6:130. 10.1186/1471-2334-6-130 PMC1564025 16914034

[B81] Külpmann R. Christiansen B. Kramer A. Lüderitz P. Pitten F.-A. Wille F. (2016). Hygiene guideline for the planning, installation, and operation of ventilation and air-conditioning systems in health-care settings–Guideline of the German Society for Hospital Hygiene (DGKH). *GMS Hyg. Infect. Control* 11:Doc03 10.3205/dgkh000263 PMC4766922 26958457

[B82] La Duc M. Kern R. Venkateswaran K. (2004). Microbial monitoring of spacecraft and associated environments. *Microb. Ecol.* 47 150–158. 10.1007/s00248-003-1012-0 14749906

[B83] La Duc M. T. Dekas A. Osman S. Moissl C. Newcombe D. Venkateswaran K. (2007). Isolation and characterization of bacteria capable of tolerating the extreme conditions of clean room environments. *Appl. Environ. Microbiol.* 73 2600–2611. 10.1128/AEM.03007-06 17308177 PMC1855582

[B84] La Duc M. T. Nicholson W. Kern R. Venkateswaran K. (2003a). Microbial characterization of the Mars Odyssey spacecraft and its encapsulation facility. *Environ. Microbiol.* 5 977–985. 10.1046/j.1462-2920.2003.00496.x 14510851

[B85] La Duc M. T. Sumner R. Pierson D. Venkat P. Venkateswaran K. (2003b). Evidence of pathogenic microbes in the International Space Station drinking water: reason for concern? *Habitation (Elmsford)* 10 39–48. 10.3727/154296604774808883 15880908

[B86] La Duc M. T. Osman S. Vaishampayan P. Piceno Y. Andersen G. Spry J. (2009). Comprehensive census of bacteria in clean rooms by using DNA microarray and cloning methods. *Appl. Environ. Microbiol.* 75 6559–6567. 10.1128/AEM.01073-09 19700540 PMC2765134

[B87] Landrin A. Bissery A. Kac G. (2005). Monitoring air sampling in operating theatres: can particle counting replace microbiological sampling? *J. Hosp. Infect.* 61 27–29. 10.1016/j.jhin.2005.03.002 16009457

[B88] Li C.-S. Hou P.-A. (2003). Bioaerosol characteristics in hospital clean rooms. *Sci. Total Environ.* 305 169–176. 10.1016/S0048-9697(02)00500-4 12670766

[B89] Lives S. S. S. (2009). *WHO Guidelines for Safe Surgery 2009.* Geneva: World Health Organization. 23762968

[B90] Livornese L. L. Dias S. Samel C. Romanowski B. Taylor S. May P. (1992). Hospital-acquired infection with vancomycin-resistant *Enterococcus faecium* transmitted by electronic thermometers. *Ann. Intern. Med.* 117 112–116. 10.7326/0003-4819-117-2-112 1605425

[B91] Lynch R. J. Englesbe M. J. Sturm L. Bitar A. Budhiraj K. Kolla S. (2009). Measurement of foot traffic in the operating room: implications for infection control. *Am. J. Med. Qual.* 24 45–52. 10.1177/1062860608326419 19139463

[B92] Mahnert A. Moissl-Eichinger C. Berg G. (2015a). Microbiome interplay: plants alter microbial abundance and diversity within the built environment. *Front. Microbiol.* 6:887 10.3389/fmicb.2015.00887 PMC4552223 26379656

[B93] Mahnert A. Vaishampayan P. Probst A. J. Auerbach A. Moissl-Eichinger C. Venkateswaran K. (2015b). Cleanroom maintenance significantly reduces abundance but not diversity of indoor microbiomes. *PLoS ONE* 10:e0134848 10.1371/journal.pone.0134848 PMC4537314 26273838

[B94] Mangram A. J. Horan T. C. Pearson M. L. Silver L. C. Jarvis W. R. Committee H. I. C. P. A. (1999). Guideline for prevention of surgical site infection, 1999. *Am. J. Infect. Control* 27 97–134. 10.1016/S0196-6553(99)70088-X 10196487

[B95] Marinella M. A. Pierson C. Chenoweth C. (1997). The stethoscope: a potential source of nosocomial infection? *Arch. Intern. Med.* 157 786–790. 10.1001/archinte.1997.00440280114010 9125011

[B96] McLeod V. (2010). *Biosafety Levels 1, 2, 3, & 4. LabManager.* Available at: http://www.labmanager.com/lab-health-and-safety/2010/12/biosafety-levels-1-2-3-4?fw1pk=2#.VVjbf5VFBxQ

[B97] Meadow J. F. Altrichter A. E. Bateman A. C. Stenson J. Brown G. Green J. L. (2015). Humans differ in their personal microbial cloud. *PeerJ* 3 e1258. 10.7717/peerj.1258 PMC4582947 26417541

[B98] Moissl C. Bruckner J. C. Venkateswaran K. (2008). Archaeal diversity analysis of spacecraft assembly clean rooms. *ISME J.* 2 115–119. 10.1038/ismej.2007.98 18180750

[B99] Moissl C. Osman S. La Duc M. T. Dekas A. Brodie E. DeSantis T. (2007). Molecular bacterial community analysis of clean rooms where spacecraft are assembled. *FEMS Microbiol. Ecol.* 61 509–521. 10.1111/j.1574-6941.2007.00360.x 17655710

[B100] Moissl-Eichinger C. (2011). Archaea in artificial environments: their presence in global spacecraft clean rooms and impact on planetary protection. *ISME J.* 5 209–219. 10.1038/ismej.2010.124 20703318 PMC3105705

[B101] Moissl-Eichinger C. Auerbach A. K. Probst A. J. Mahnert A. Tom L. Piceno Y. (2015). Quo vadis? Microbial profiling revealed strong effects of cleanroom maintenance and routes of contamination in indoor environments. *Sci. Rep.* 5:9156 10.1038/srep09156 PMC4361859 25778463

[B102] Moissl-Eichinger C. Cockell C. Rettberg P. (2016). Venturing into new realms? Microorganisms in space. *FEMS Microbiol. Rev.* 40 722–737. 10.1093/femsre/fuw015 27354346

[B103] Moissl-Eichinger C. Pukall R. Probst A. J. Stieglmeier M. Schwendner P. Mora M. (2013). Lessons learned from the microbial analysis of the Herschel spacecraft during assembly, integration, and test operations. *Astrobiology* 13 1125–1139. 10.1089/ast.2013.1024 24313230

[B104] Moissl-Eichinger C. Rettberg P. Pukall R. (2012). The first collection of spacecraft-associated microorganisms: a public source for extremotolerant microorganisms from spacecraft assembly clean rooms. *Astrobiology* 12 1024–1034. 10.1089/ast.2012.0906 23121015

[B105] Mord I. S. S. (2009). SSP 50260: ISS medical operations requirement document. *Houston* 307:22.

[B106] Myers M. G. (1978). Longitudinal evaluation of neonatal nosocomial infections: association of infection with a blood pressure cuff. *Pediatrics* 61 42–45. 263872

[B107] Naesens R. Jeurissen A. Vandeputte C. Cossey V. Schuermans A. (2009). Washing toys in a neonatal intensive care unit decreases bacterial load of potential pathogens. *J. Hosp. Infect.* 71 197–198. 10.1016/j.jhin.2008.10.018 19100660

[B108] Napoli C. Marcotrigiano V. Montagna M. (2012). Air sampling procedures to evaluate microbial contamination: a comparison between active and passive methods in operating theatres. *BMC Public Health* 12:594 10.1186/1471-2458-12-594 PMC3444341 22853006

[B109] Nicholson W. L. Schuerger A. C. Race M. S. (2009). Migrating microbes and planetary protection. *Trends Microbiol.* 17 389–392. 10.1016/j.tim.2009.07.001 19726193

[B110] Nitta K. (1999). Basic design concept of closed ecology experiment facilities. *Adv. Space Res.* 24 343–350. 10.1016/S0273-1177(99)00322-1 11542543

[B111] Novikova N. (2004). Review of the knowledge of microbial contamination of the Russian manned spacecraft. *Microb. Ecol.* 47 127–132. 10.1007/s00248-003-1055-2 14994178

[B112] Novikova N. De Boever P. Poddubko S. Deshevaya E. Polikarpov N. Rakova N. (2006). Survey of environmental biocontamination on board the International Space Station. *Res. Microbiol.* 157 5–12. 10.1016/j.resmic.2005.07.010 16364606

[B113] Novikova N. D. Polikarpov N. A. Poddubko S. V. Deshevaya E. A. (2001). *The Results of Microbiological Research of Environmental Microflora of Orbital Station Mir.* Warrendale, PA: SAE International.

[B114] Nseir S. Blazejewski C. Lubret R. Wallet F. Courcol R. Durocher A. (2011). Risk of acquiring multidrug-resistant Gram-negative bacilli from prior room occupants in the intensive care unit. *Clin. Microbiol. Infect.* 17 1201–1208. 10.1111/j.1469-0691.2010.03420.x 21054665

[B115] Oberauner L. Zachow C. Lackner S. Högenauer C. Smolle K.-H. Berg G. (2013). The ignored diversity: complex bacterial communities in intensive care units revealed by 16S pyrosequencing. *Sci. Rep.* 3:1413 10.1038/srep01413 PMC3593336 23475210

[B116] Ott M. Pierson D. Shirakawa M. Tanigaki F. Hida M. Yamazaki T. (2014). Space habitation and microbiology: status and roadmap of space agencies. *Microbes Environ.* 29 239–242. 10.1264/jsme2.ME2903rh 25130884 PMC4159034

[B117] Parfrey L. W. Walters W. A. Knight R. (2011). Microbial eukaryotes in the human microbiome: ecology, evolution, and future directions. *Front. Cell Infect. Microbiol.* 2:153 10.3389/fmicb.2011.00153 PMC3135866 21808637

[B118] Pasquarella C. Pitzurra O. Savino A. (2000). The index of microbial air contamination. *J. Hosp. Infect.* 46 241–256. 10.1053/jhin.2000.0820 11170755

[B119] Passaretti C. L. Otter J. A. Reich N. G. Myers J. Shepard J. Ross T. (2013). An evaluation of environmental decontamination with hydrogen peroxide vapor for reducing the risk of patient acquisition of multidrug-resistant organisms. *Clin. Infect. Dis.* 56 27–35. 10.1093/cid/cis839 23042972

[B120] Peleg A. Y. Hooper D. C. (2010). Hospital-acquired infections due to gram-negative bacteria. *N. Engl. J. Med.* 362 1804–1813. 10.1056/NEJMra0904124 20463340 PMC3107499

[B121] Penders J. Thijs C. Vink C. Stelma F. F. Snijders B. Kummeling I. (2006). Factors influencing the composition of the intestinal microbiota in early infancy. *Pediatrics* 118 511–521. 10.1542/peds.2005-2824 16882802

[B122] Perez E. Williams M. Jacob J. T. Reyes M. D. Tejedor S. C. Steinberg J. P. (2014). Microbial biofilms on needleless connectors for central venous catheters: comparison of standard and silver-coated devices collected from patients in an acute care hospital. *J. Clin. Microbiol.* 52 823–831. 10.1128/JCM.02220-13 24371233 PMC3957745

[B123] Pham T. A. N. Lawley T. D. (2014). Emerging insights on intestinal dysbiosis during bacterial infections. *Curr. Opin. Microbiol.* 17 67–74. 10.1016/j.mib.2013.12.002 24581695 PMC3969284

[B124] Pierson D. L. (2001). Microbial contamination of spacecraft. *Gravit. Space Biol. Bull.* 14 1–6. 11865864

[B125] Pittet D. Allegranzi B. Sax H. Dharan S. Pessoa-Silva C. L. Donaldson L. (2006). Evidence-based model for hand transmission during patient care and the role of improved practices. *Lancet Infect. Dis.* 6 641–652. 10.1016/S1473-3099(06)70600-4 17008173

[B126] Powers E. M. (1965). *Microbial Profile of Laminar Flow Clean Rooms.* Technical Report Greenbelt, MD: NASA Goddard Space Flight Center.

[B127] Poza M. Gayoso C. Gómez M. J. Rumbo-Feal S. Tomás M. Aranda J. (2012). Exploring bacterial diversity in hospital environments by GS-FLX Titanium pyrosequencing. *PLoS ONE* 7:e44105 10.1371/journal.pone.0044105 PMC3430676 22952889

[B128] Probst A. Vaishampayan P. Osman S. Moissl-Eichinger C. Andersen G. L. Venkateswaran K. (2010). Diversity of anaerobic microbes in spacecraft assembly clean rooms. *Appl. Environ. Microbiol.* 76 2837–2845. 10.1128/AEM.02167-09 20228115 PMC2863428

[B129] Probst A. J. Auerbach A. K. Moissl-Eichinger C. (2013). Archaea on human skin. *PLoS ONE* 8:e65388 10.1371/journal.pone.0065388 PMC3680501 23776475

[B130] Puleo J. Fields N. Bergstrom S. Oxborrow G. Stabekis P. Koukol R. (1977). Microbiological profiles of the Viking spacecraft. *Appl. Environ. Microbiol.* 33 379–384. 848957 10.1128/aem.33.2.379-384.1977 PMC170694

[B131] Qian J. Hospodsky D. Yamamoto N. Nazaroff W. W. Peccia J. (2012). Size-resolved emission rates of airborne bacteria and fungi in an occupied classroom. *Indoor Air* 22 339–351. 10.1111/j.1600-0668.2012.00769.x 22257156 PMC3437488

[B132] Raschka S. Dempster L. Bryce E. (2013). Health economic evaluation of an infection prevention and control program: are quality and patient safety programs worth the investment? *Am. J. Infect. control* 41 773–777. 10.1016/j.ajic.2012.10.026 23993762

[B133] Richmond Y. S. McKinney R. W. (1999). *Biosafety in Microbiological and Biomedical Laboratories (BMCL).* Washington, DC: US Government Printing Office.

[B134] Ruiz-Calderon J. F. Cavallin H. Song S. J. Novoselac A. Pericchi L. R. Hernandez J. N. (2016). Walls talk: microbial biogeography of homes spanning urbanization. *Sci. Adv.* 2:e1501061 10.1126/sciadv.1501061 PMC4758746 26933683

[B135] Russotto V. Cortegiani A. Raineri S. M. Giarratano A. (2015). Bacterial contamination of inanimate surfaces and equipment in the intensive care unit. *J. Intensive Care* 3:54 10.1186/s40560-015-0120-5 PMC4676153 26693023

[B136] Rutala W. A. Weber D. J. Centers for Disease Control (2008). *Guideline for Disinfection and Sterilization in Healthcare Facilities, 2008.* Atlanta, GA: Centers for Disease Control.

[B137] Safdar N. Drayton J. Dern J. Warrack S. Duster M. Schmitz M. (2012). Telemetry leads harbor nosocomial pathogens. *Int. J. Infect. Control* 8 10–12. 10.3396/ijic.v8i2.012.12

[B138] Salassa T. E. Swiontkowski M. F. (2014). Surgical attire and the operating room: role in infection prevention. *J. Bone Joint Surg. Am.* 96 1485–1492. 10.2106/JBJS.M.01133 25187588

[B139] Salgado C. D. Sepkowitz K. A. John J. F. Cantey J. R. Attaway H. H. Freeman K. D. (2013). Copper surfaces reduce the rate of healthcare-acquired infections in the intensive care unit. *Infect. Control Hosp. Epidemiol.* 34 479–486. 10.1086/670207 23571364

[B140] Satoh K. Nishiyama Y. Yamazaki T. Sugita T. Tsukii Y. Takatori K. (2011). Microbe-I: fungal biota analyses of the Japanese experimental module KIBO of the International Space Station before launch and after being in orbit for about 460 days. *Microbiol. Immunol.* 55 823–829. 10.1111/j.1348-0421.2011.00386.x 21950271

[B141] Scaltriti S. Cencetti S. Rovesti S. Marchesi I. Bargellini A. Borella P. (2007). Risk factors for particulate and microbial contamination of air in operating theatres. *J. Hosp. Infect.* 66 320–326. 10.1016/j.jhin.2007.05.019 17655973

[B142] Schabrun S. Chipchase L. Rickard H. (2006). Are therapeutic ultrasound units a potential vector for nosocomial infection? *Physiother. Res. Int.* 11 61–71. 10.1002/pri.329 16808087

[B143] Schmidt M. G. von Dessauer B. Benavente C. Benadof D. Cifuentes P. Elgueta A. (2015). Copper surfaces are associated with significantly lower concentrations of bacteria on selected surfaces within a pediatric intensive care unit. *Am. J. Infect. Control* 44 203–209. 10.1016/j.ajic.2015.09.008 26553403

[B144] Schröder C. Schwab F. Behnke M. Breier A. C. Maechler F. Piening B. (2015). Epidemiology of healthcare associated infections in Germany: nearly 20 years of surveillance. *Int. J. Med. Microbiol.* 305 799–806. 10.1016/j.ijmm.2015.08.034 26358916

[B145] Seal D. V. Clark R. P. (1990). Electronic particle counting for evaluating the quality of air in operating theatres: a potential basis for standards? *J. Appl. Bacteriol.* 68 225–230. 10.1111/j.1365-2672.1990.tb02568.x 2341326

[B146] Sehulster L. Chinn R. Y. W. Arduino M. J. Carpenter J. Donlan R. Ashford D. (2003). Guidelines for environmental infection control in health-care facilities. Recommendations of CDC and the Healthcare Infection Control Practices Advisory Committee (HICPAC). *MMWR Recomm. Rep.* 52 1–42. 12836624

[B147] Semmelweis I. (1988). “The etiology, concept, and prophylaxis of childbed fever,” in *The Challenge of Epidemiology. Issues and Selected Readings* eds Buck C. Llopis A. Najera E. Terris M. (Washington, DC: World Health Organization) 46–59.

[B148] Shin H. Pei Z. Martinez K. A. Rivera-Vinas J. I. Mendez K. Cavallin H. (2015). The first microbial environment of infants born by C-section: the operating room microbes. *Microbiome* 3:59 10.1186/s40168-015-0126-1 PMC4665759 26620712

[B149] Siegel J. D. Rhinehart E. Jackson M. Chiarello L. (2007). 2007 guideline for isolation precautions: preventing transmission of infectious agents in health care settings. *Am. J. Infect. Control* 35 S65–S164. 10.1016/j.ajic.2007.10.007 18068815 PMC7119119

[B150] Singh N. Léger M.-M. Campbell J. Short B. Campos J. M. (2005). Control of vancomycin-resistant enterococci in the neonatal intensive care unit. *Infect. Control Hosp. Epidemiol.* 26 646–649. 10.1086/502595 16092746

[B151] Sonnenfeld G. Shearer W. T. (2002). Immune function during space flight. *Nutrition* 18 899–903. 10.1016/S0899-9007(02)00903-6 12361785

[B152] Spagnolo A. Ottria G. Amicizia D. Perdelli F. Cristina M. L. (2013). Operating theatre quality and prevention of surgical site infections. *J. Prev. Med. Hyg.* 54 131–137. 24783890 PMC4718372

[B153] Stieglmeier M. Rettberg P. Barczyk S. Bohmeier M. Pukall R. Wirth R. (2012). Abundance and diversity of microbial inhabitants in European spacecraft-associated clean rooms. *Astrobiology* 12 572–585. 10.1089/ast.2011.0735 22794299

[B154] Stieglmeier M. Wirth R. Kminek G. Moissl-Eichinger C. (2009). Cultivation of anaerobic and facultatively anaerobic bacteria from spacecraft-associated clean rooms. *Appl. Environ. Microbiol.* 75 3484–3491. 10.1128/AEM.02565-08 19363082 PMC2687301

[B155] Stocks G. W. Self S. D. Thompson B. Adame X. A. O’Connor D. P. (2010). Predicting bacterial populations based on airborne particulates: a study performed in nonlaminar flow operating rooms during joint arthroplasty surgery. *Am. J. Infect. Control* 38 199–204. 10.1016/j.ajic.2009.07.006 19913327

[B156] Stover B. H. Shulman S. T. Bratcher D. F. Brady M. T. Levine G. L. Jarvis W. R. (2001). Nosocomial infection rates in US children’s hospitals’ neonatal and pediatric intensive care units. *Am. J. Infect. Control* 29 152–157. 10.1067/mic.2001.115407 11391276

[B157] Sun Y. Xie B. Wang M. Dong C. Du X. Fu Y. (2016). Microbial community structure and succession of airborne microbes in closed artificial ecosystem. *Ecol. Eng.* 88 165–176. 10.1016/j.ecoleng.2015.12.013

[B158] Taylor P. W. (2015). Impact of space flight on bacterial virulence and antibiotic susceptibility. *Infect. Drug Res.* 8 249–262. 10.2147/IDR.S67275 PMC4524529 26251622

[B159] Thomas M. Sanborn M. D. Couldry R. (2005). IV admixture contamination rates: traditional practice site versus a class 1000 cleanroom. *Am. J. Health Syst. Pharm.* 62 2386–2392. 10.2146/ajhp050078 16278330

[B160] Touati A. Achour W. Cherif A. Hmida H. B. Afif F. B. Jabnoun S. (2009). Outbreak of *Acinetobacter baumannii* in a neonatal intensive care unit: antimicrobial susceptibility and genotyping analysis. *Ann. Epidemiol.* 19 372–378. 10.1016/j.annepidem.2009.03.010 19364663

[B161] Triassi M. Di Popolo A. D’Alcalà G. R. Albanese Z. Cuccurullo S. Montegrosso S. (2006). Clinical and environmental distribution of *Legionella pneumophila* in a university hospital in Italy: efficacy of ultraviolet disinfection. *J. Hosp. Infect.* 62 494–501. 10.1016/j.jhin.2005.09.029 16455159

[B162] Tringe S. G. Hugenholtz P. (2008). A renaissance for the pioneering 16S rRNA gene. *Curr. Opin. Microbiol.* 11 442–446. 10.1016/j.mib.2008.09.011 18817891

[B163] Unahalekhaka A. (2011). “Epidemiology of health care-associated infections,” in *IFIC Basic Concepts of Infection Control* eds Friedman C. Newsom W. (Portadown: International Federation of Infection Control) 27–40.

[B164] Urrea M. Pons M. Serra M. Latorre C. Palomeque A. (2003). Prospective incidence study of nosocomial infections in a pediatric intensive care unit. *Pediatr. Infect. Dis. J.* 22 490–493. 10.1097/01.inf.0000069758.00079.d3 12799503

[B165] US Department of Health and Human Services (2009). *HHS Publication No.(CDC) 21-11: Biosafety in Microbiological and Biomedical Laboratories.* Washington, DC: US Government Printing Office.

[B166] Vaishampayan P. Probst A. J. La Duc M. T. Bargoma E. Benardini J. N. Andersen G. L. (2013). New perspectives on viable microbial communities in low-biomass cleanroom environments. *ISME J.* 7 312–324. 10.1038/ismej.2012.114 23051695 PMC3554398

[B167] Van den Berg R. Claahsen H. Niessen M. Muytjens H. Liem K. Voss A. (2000). *Enterobacter cloacae* outbreak in the NICU related to disinfected thermometers. *J. Hosp. Infect.* 45 29–34. 10.1053/jhin.1999.0657 10917779

[B168] van Elsas J. D. Chiurazzi M. Mallon C. A. Elhottovā D. Krištůfek V. Salles J. F. (2012). Microbial diversity determines the invasion of soil by a bacterial pathogen. *Proc. Natl. Acad. Sci. U.S.A.* 109 1159–1164. 10.1073/pnas.1109326109 22232669 PMC3268289

[B169] Van Houdt R. Mijnendonckx K. Leys N. (2012). Microbial contamination monitoring and control during human space missions. *Planet. Space Sci.* 60 115–120. 10.1016/j.pss.2011.09.001

[B170] Venkatesan A. Kansal S. Patel S. S. Akulwar S. K. (2015). The role of hand hygiene and mobile phones in transmitting hospital acquired infection. *Int. J. Biomed. Adv. Res.* 6 435–437.

[B171] Venkateswaran K. La Duc M. T. Horneck G. (2014a). Microbial existence in controlled habitats and their resistance to space conditions. *Microbes Environ.* 29 243–249. 10.1264/jsme2.ME14032 25130881 PMC4159035

[B172] Venkateswaran K. Vaishampayan P. Benardini J. N. III Rooney A. P. Spry J. A. (2014b). Deposition of extreme-tolerant bacterial strains isolated during different phases of Phoenix spacecraft assembly in a public culture collection. *Astrobiology* 14 24–26. 10.1089/ast.2013.0978 24392704

[B173] Venkateswaran K. Vaishampayan P. Cisneros J. Pierson D. L. Rogers S. O. Perry J. (2014c). International Space Station environmental microbiome—microbial inventories of ISS filter debris. *Appl. Microbiol. Biotechnol.* 98 6453–6466. 10.1007/s00253-014-5650-6 24695826

[B174] Verde S. C. Almeida S. M. Matos J. Guerreiro D. Meneses M. Faria T. (2015). Microbiological assessment of indoor air quality at different hospital sites. *Res. Microbiol.* 166 557–563. 10.1016/j.resmic.2015.03.004 25869221

[B175] Vesley D. Keenan K. Halbert M. (1966). Effect of time and temperature in assessing microbial contamination on flat surfaces. *Appl. Microbiol.* 14 203–205. 5335385 10.1128/am.14.2.203-205.1966 PMC546650

[B176] Vesper S. J. Wong W. Kuo C. M. Pierson D. L. (2008). Mold species in dust from the International Space Station identified and quantified by mold-specific quantitative PCR. *Res. Microbiol.* 159 432–435. 10.1016/j.resmic.2008.06.001 18602989

[B177] Vickery K. Deva A. Jacombs A. Allan J. Valente P. Gosbell I. (2012). Presence of biofilm containing viable multiresistant organisms despite terminal cleaning on clinical surfaces in an intensive care unit. *J. Hosp. Infect.* 80 52–55. 10.1016/j.jhin.2011.07.007 21899921

[B178] Vincent J. L. Bihari D. J. Suter P. M. Bruining H. A. White J. Nicolas-Chanoin M. H. (1995). The prevalence of nosocomial infection in intensive care units in Europe. Results of the European Prevalence of Infection in Intensive Care (EPIC) Study. EPIC International Advisory Committee. *JAMA* 274 639–644. 10.1001/jama.274.8.639 7637145

[B179] Wan G.-H. Chung F.-F. Tang C.-S. (2011). Long-term surveillance of air quality in medical center operating rooms. *Am. J. Infect. Control* 39 302–308. 10.1016/j.ajic.2010.07.006 21256628

[B180] Weber D. J. Rutala W. A. Miller M. B. Huslage K. Sickbert-Bennett E. (2010). Role of hospital surfaces in the transmission of emerging health care-associated pathogens: norovirus, Clostridium difficile, and *Acinetobacter* species. *Am. J. Infect. Control* 38 S25–S33. 10.1016/j.ajic.2010.04.196 20569853

[B181] Weinmaier T. Probst A. J. Duc M. T. Ciobanu D. Cheng J.-F. Ivanova N. (2015). A viability-linked metagenomic analysis of cleanroom environments: eukarya, prokaryotes, and viruses. *Microbiome* 3:62 10.1186/s40168-015-0129-y PMC4672508 26642878

[B182] Wertheim H. F. Melles D. C. Vos M. C. van Leeuwen W. van Belkum A. Verbrugh H. A. (2005). The role of nasal carriage in *Staphylococcus aureus* infections. *Lancet Infect. Dis.* 5 751–762. 10.1016/S1473-3099(05)70295-4 16310147

[B183] Whitfield W. (1964). Ultra-clean room. US 3158457A

[B184] Whyte W. (1999). *Cleanroom design.* West Sussex: John Wiley & Sons Ltd.

[B185] Whyte W. (2001). *Cleanroom Technology-Fundamentals of Design. Testing, and Operation.* West Sussex: Johnson Wiley & Sons.

[B186] Whyte W. (2010). *Cleanroom Technology: Fundamentals of Design, Testing and Operation* 2nd Edn West Sussex: John Wiley & Sons, Ltd.

[B187] Whyte W. Eaton T. (2004a). Microbial risk assessment in pharmaceutical cleanrooms. *Eur. J. Parenteral Pharm. Sci.* 9 16–23. 10.5731/pdajpst.2011.00693

[B188] Whyte W. Eaton T. (2004b). Microbiological contamination models for use in risk assessment during pharmaceutical production. *Eur. J. Parenteral Pharm. Sci.* 9 11–15.

[B189] Whyte W. Hejab M. (2007). Particle and microbial airborne dispersion from people. *Eur. J. Parenteral Pharm. Sci.* 12 39–46.

[B190] Wilson D. E. Chosewood L. C. (2007). “Biosafety in microbiological and biomedical laboratories,” in *US Department of Health and Human Services, CDC/NIH* 5th Edn eds Chosewood L. C. Wilson D. E. (Washington, DC: US Government Printing Office).

[B191] Wilson J. Ott C. Zu Bentrup K. H. Ramamurthy R. Quick L. Porwollik S. (2007). Space flight alters bacterial gene expression and virulence and reveals a role for global regulator Hfq. *Proc. Natl. Acad. Sci. U.S.A.* 104 16299–16304. 10.1073/pnas.0707155104 17901201 PMC2042201

[B192] Wolcott R. D. Gontcharova V. Sun Y. Zischakau A. Dowd S. E. (2009). Bacterial diversity in surgical site infections: not just aerobic cocci any more. *J. Wound Care* 18 317–323. 10.12968/jowc.2009.18.8.43630 19862869

[B193] World Health Organization (2005). *Enhancement of Laboratory Biosafety. World Health Assembly resolution WHA58.29.* Geneva: World Health Organization.

[B194] Wu G.-F. Liu X.-H. (2007). Characterization of predominant bacteria isolates from clean rooms in a pharmaceutical production unit. *J. Zhejiang Univ. Sci. B* 8 666–672. 10.1631/jzus.2007.B0666 17726748 PMC1963433

[B195] Yamaguchi N. Ichijo T. Nasu M. (2016). Bacterial Monitoring in the International Space Station-&ldquo;Kibo&rdquo; based on rRNA gene sequence. *Trans. Jpn Soc. Aeronaut. Space Sci. Aerosp. Technol. Jpn* 14 Pp_1–Pp_4. 10.2322/tastj.14.Pp_1

